# Transforming the Poison Effects of Water Vapor into Benefits Over Adjustable Dual Acid Sites for Stable Plasma‐Catalysis

**DOI:** 10.1002/advs.202502123

**Published:** 2025-04-26

**Authors:** Si Chen, Sibo Zhang, Lu Fang, Yan Yang, Chenyuan Zhu, Xinyi Dai, Zhengjun Gong, Fan Dong

**Affiliations:** ^1^ Research Center for Carbon‐Neutral Environmental & Energy Technology Institute of Fundamental and Frontier Sciences University of Electronic Science and Technology of China Chengdu 611731 China; ^2^ Huzhou Key Laboratory of Smart and Clean Energy Yangtze Delta Region Institute (Huzhou) University of Electronic Science and Technology of China Huzhou 313000 China; ^3^ School of Chemical Engineering and Light Industry Guangdong University of Technology Guangzhou 510006 China; ^4^ School of Environmental Science and Engineering Southwest Jiaotong University Chengdu 610031 China

**Keywords:** co‐decomposition, ethyl acetate degradation, H_2_O utilization, O_3_, plasma‐catalysis, Y_x_Mn_y_O_x+2y_

## Abstract

Developing a new strategy to address water vapor poisoning is crucial for catalysts in real‐working conditions. Except for the traditional thinking of resistance enhancement, a reverse idea is proposed herein of utilizing the inevitable H_2_O, converting it to active ·OH to enhance the overall performance, with the help of O_3_ and high energy electrons (e*) in plasma. Dual active sites of Lewis acid (Y^3+^) and Mn on Y_x_Mn_y_O_x+2y_ catalyst promote the co‐adsorption of H_2_O and O_3_, and the dissociation of H_2_O to surface hydroxyl species (*OH). A new OH‐accompanied pathway for O_3_ decomposition is formed and a new intermediate species (*OOH) with a lower energy barrier (0.77 eV lower than traditional *O_2_^2−^) is detected, in which e* in plasma can further accelerate its desorption. Thereafter, abundant active ·OH are generated and work for pollutants degradation, achieving 99.78% ethyl acetate (EA) degradation and 97.36% mineralization rate on the surface of YMO (1:2) under humid environment, with excellent long‐term stability. The changed activation site of C─O bond in EA, different by‐products, and reaction pathways are also analyzed. This active species regulation strategy transforms the traditional poison effects of water vapor into great benefits, paving the way for broader catalyst applications free of water vapor.

## Graphical abstract

Y_x_Mn_y_O_x+2y_ catalyst provides dual active sites of Lewis acid (Y^3+^) and Mn for the co‐adsorption of H_2_O and O_3_, and the fast dissociation of adsorbed H_2_O to surface *OH. Then, a newly OH‐accompanied pathway for O_3_ decomposition is formed, transforming H_2_O to active ·OH radical, with the assistance of high energy electrons in the plasma. This realizes superstable plasma‐catalytic VOCs degradation under humidity, transforming H_2_O‐poison into benefits.

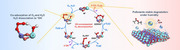

## 1 Introduction

Catalysts deactivation by the inevitable water vapor (H_2_O) in real‐working conditions is a widely encountered issue in heterogeneous catalysis process, leading to production shutdown, profit losses, or violation of emission standards^[^ ^1^ ^]^ H_2_O will occupy the active sites on the catalysts surface and hard to desorb (the formed surface hydroxyl groups (*OH) exhibit great thermal stability below 250 °C), resulting in irreversible deactivation of catalysts.^[^ ^2^ ^]^ For decades, intensive efforts have been devoted to addressing this issue in many industrial reactions such as selective catalytic reduction of NOx (SCR),^[^ ^3^ ^]^ catalytic oxidation of volatile organic compounds (VOCs),^[^ ^4^ ^]^ chemical synthesis,^[^ ^5^ ^]^ etc. Strategies like active site protection,^[^ ^6^ ^]^ hydrophobic layer construction,^[^ ^7^ ^]^ redox properties enhancement,^[^ ^8^ ^]^ etc. have been developed to improve the water tolerance of catalysts, and great effects have been achieved. However, those do not settle the problems fundamentally but only prolong the lifetime of catalysts to a certain degree.

**Figure 1:**
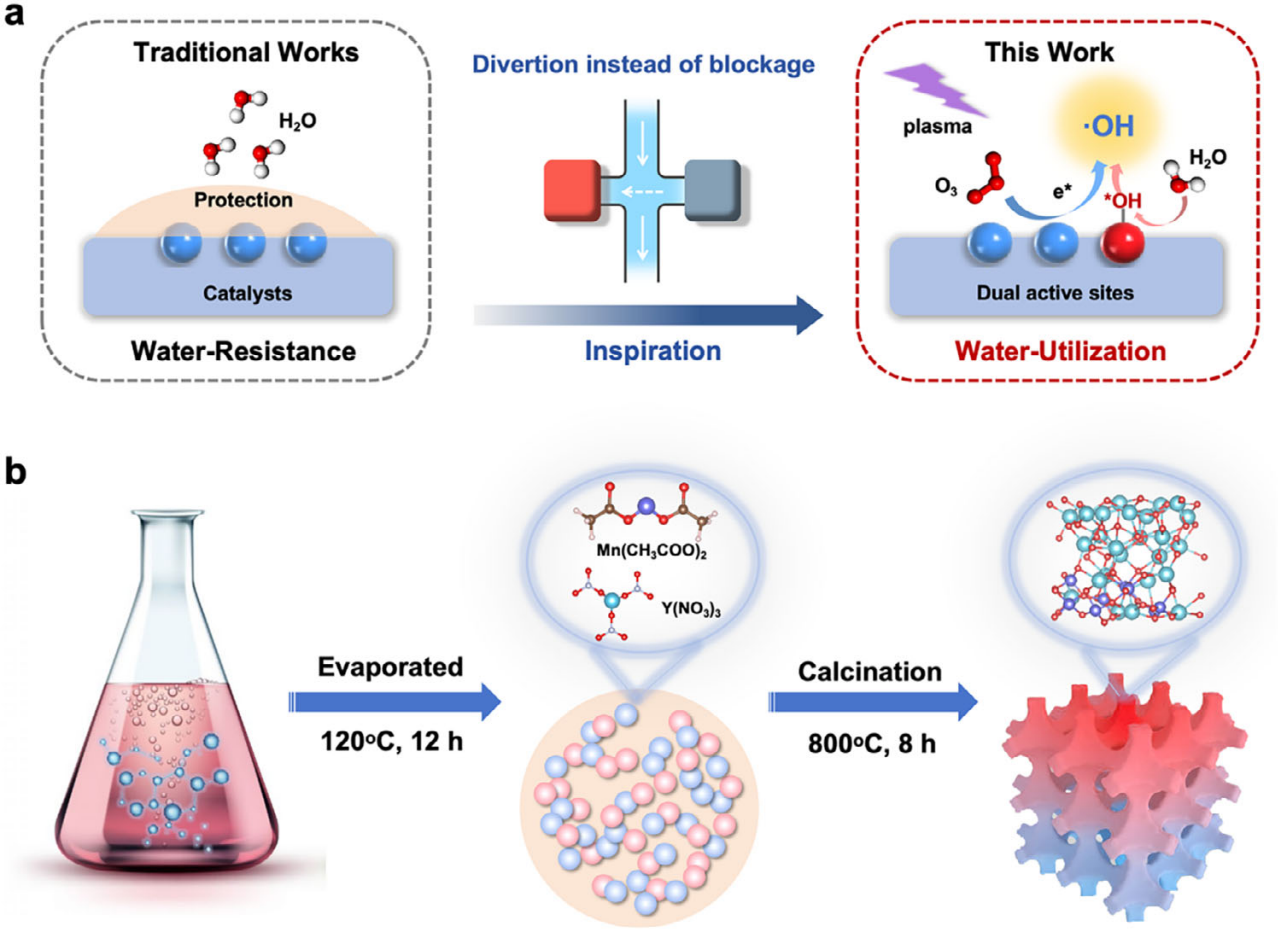
a) Schematic of the addressment of water vapor: traditional versus utilized strategy. b) The synthesis process of Y_x_Mn_y_O_x+2y_ catalysts.

Inspired by the China traditional fable of “Yu the great harnesses the flood”, if a thing is inevitable, try to use it, instead of escape. Significantly yet often overlooked, hydroxyl radicals (·OH) with super oxidizability are just derived from H_2_O.^[^ ^9^ ^]^ Imagine that if the surface adsorbed H_2_O can be dissociated and form ·OH to participate in reactions with the assistance of a certain external force, catalysts’ deactivation from H_2_O will be smoothly solved as well as enhance the overall oxidation performance, converting the disadvantages into advantages (**Figure** **1**a).

Plasma‐catalysis technology containing extensive high‐energy electrons (e*) and self‐generated ozone (O_3_) provides the chance.^[^ ^10^ ^]^ As widely reported, water vapor can decompose and form ·OH through the collision reaction with e* in plasma.^[^ ^11^ ^]^ Meanwhile, H_2_O/*OH can react with the intermediate active species derived from O_3_ catalytic decomposition to form ·OH and other active radicals like O_2_·^−^, and ^1^O_2_, exhibiting excellent oxidizing ability.^[^ ^12^ ^]^ As demonstrated by Huang's group,^[^ ^13^ ^]^ the *OH on the surface of catalysts greatly improved the reactive oxygen species generation especially ·OH during the O_3_ catalytically decomposition, boosting VOCs degradation.

Significantly, however, in the gas‐solid reaction process, only the pre‐formed *OH can be converted to ·OH during O_3_ catalytic decomposition, and the competitive adsorption between O_3_ and gaseous H_2_O molecules on catalysts like the classic MnO_x_ is still ubiquitous,^[^ ^14^ ^]^ severely influencing its original utilizing rate of O_3_. Zhu et al.^[^ ^15^ ^]^ also emphasized the critical role of H_2_O dissociation for the O_3_ degradation under humidity. More seriously, free water vapor would cause the quenching of e* in plasma, restraining the overall performance.^[^ ^16^ ^]^ That is, the real issue is to quickly anchor the H_2_O molecules onto the surface of a catalyst and dissociate to *OH species, on the premise of keeping the original catalytic properties.

Constructing an additional site for the adsorption and activation of water vapor may possibly be a solution, to quickly capture the H_2_O molecules on the catalyst surface and transform to ·OH under the attack of e* and the O_3_ catalytic decomposition in plasma. We herein prepared a series of yttrium (Y)‐doped MnO_x_ (YMO) catalysts with dual active sites, where Mn sites are responsible for O_3_ catalytic decomposition and Y sites for the adsorption and activation of water vapor thanks to its special water affinity and Lewis acid property.^[^ ^17^ ^]^ The performance of water utilization and O_3_ utilization are dynamically adjusted via the molar ratio of Y and Mn, and water vapor can be effectively anchored on the Y sites and dissociated to form *OH. Then a new OH‐accompanied pathway for O_3_ degradation is formed, and abundant active ·OH is generated for typical VOCs degradation. As acknowledged, plasma‐catalysis technology is suitable and widely used for the elimination of VOCs, characterized by low‐concentration and high‐volume, emitted from chemical industries such as coating, printing, metallurgy, pharmaceuticals, etc.^[^ ^18^ ^]^ In these industries, ethyl acetate (EA) is widely used as an organic solvent due to its good solubility and fast evaporation rate,^[^ ^19^ ^]^ and therefore, EA is used as the typical VOCs in this study. The detailed degradation pathway and key intermediates are also illustrated. It is expected to provide a reverse thinking to address the ubiquitous issue of water poisoning, converting the poison effects into benefits.

## 2 Results and Discussion

### 2.1 Y_x_Mn_y_O_x+2y_ Catalysts Fabrication and Dual Active Sites Identification

**Figure 2:**
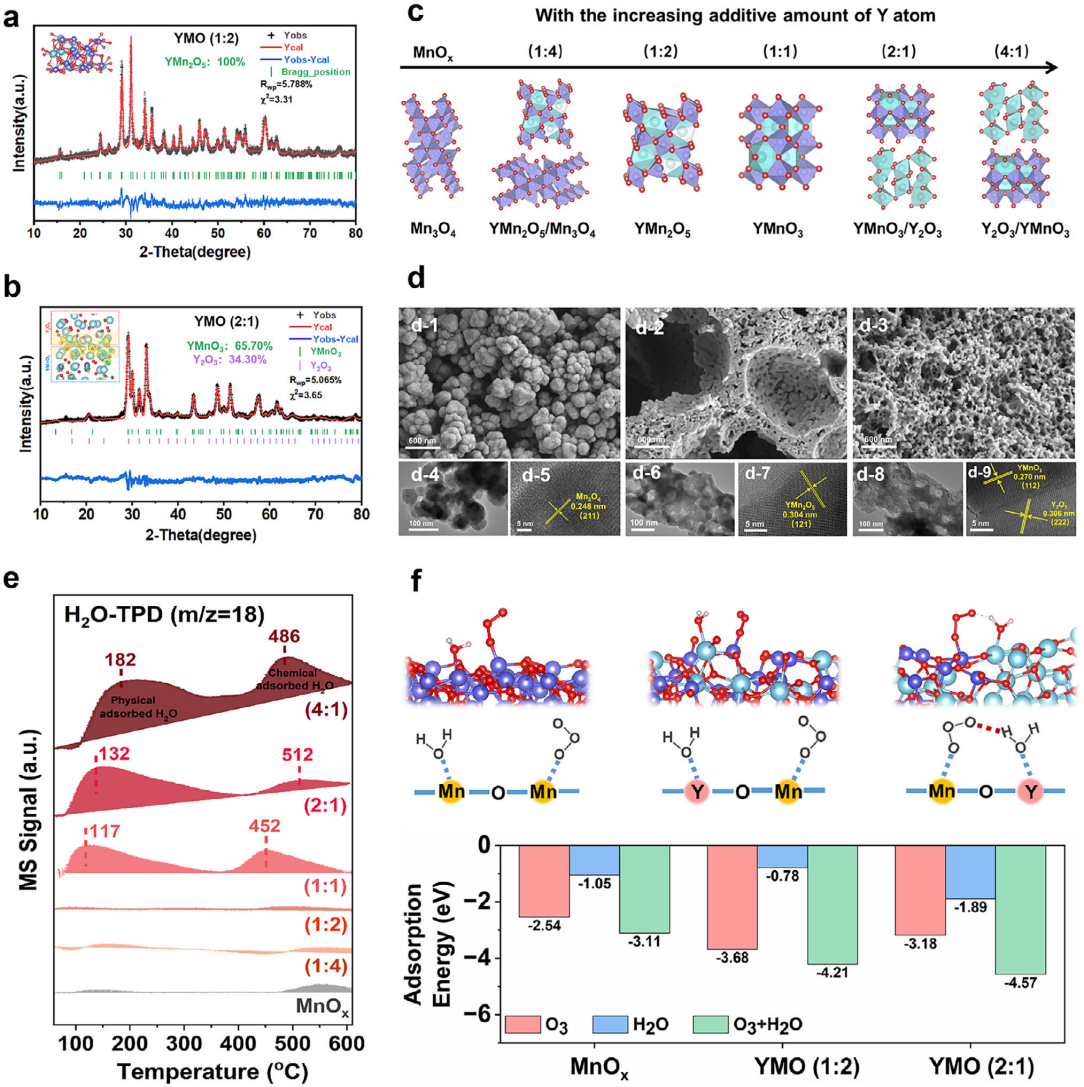
XRD Rietveld refinement of a) YMO (1:2), and b) YMO (2:1) (Yobs: observed Bragg position, Ycal: calculated Bragg position, Yobs‐Ycal: difference), and the inset in Figure 1b: Charge difference distribution (DIFF) between YMnO_3_ and Y_2_O_3_ in YMO (2:1), charge accumulation depicted in yellow and depletion in blue, and the isosurface set to 0.005 eV Å^−3^ (Y: sky‐blue, O: red, Mn: violet). c) A schematic diagram of the crystal structure of the samples. SEM and TEM image of (d‐1), (d‐4), and (d‐5) for MnO_x_, (d‐2), (d‐6), and (d‐7) for YMO (1:2), and (d‐3), (d‐8) and (d‐9) for YMO (2:1). e) H_2_O‐TPD profiles of the as‐prepared samples (Enlarged view for MnO_x_, YMO (1:4) and YMO (1:2) are shown in Figure S5, Supporting Information). f) The optimized structure of co‐adsorption of H_2_O and O_3_ molecules adsorbed on MnO_x_, YMO (1:2), and YMO (2:1), and their adsorption energy.

**Figure 3:**
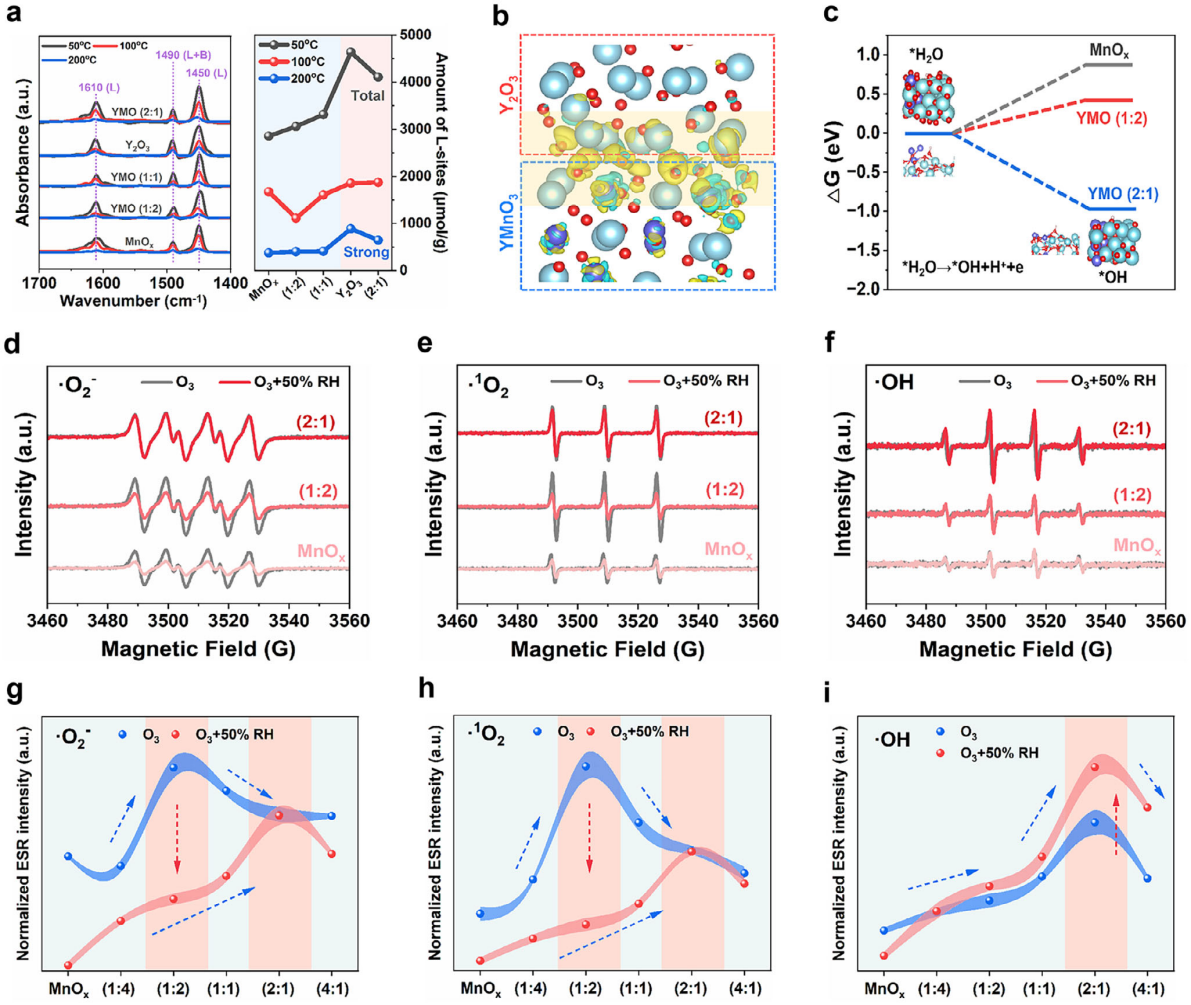
a) Pyridine‐IR spectra of the as‐prepared samples at 50 °C, 100 °C, and 200 °C, and the amount of the Lewis‐acid sites over these two samples. b) Charge difference distribution (DIFF) between YMnO_3_ and Y_2_O_3_ in YMO (2:1), charge accumulation depicted in yellow and depletion in blue, and the isosurface set to 0.005 eV Å‐3 (Y: sky‐blue, O: red, Mn: violet). c) The Gibbs free energy of reaction with *OH formation on the surface of as prepared samples. ESR signals of (d) ·O_2_^−^, (e) ·^1^O_2,_ and f) ·OH of different samples with O_3_ purging at 30 °C under dry and humid conditions. g–i) Normalized intensity of ESR signals for different samples with O_3_ purging at 30 °C under dry and humid conditions.

**Figure 4:**
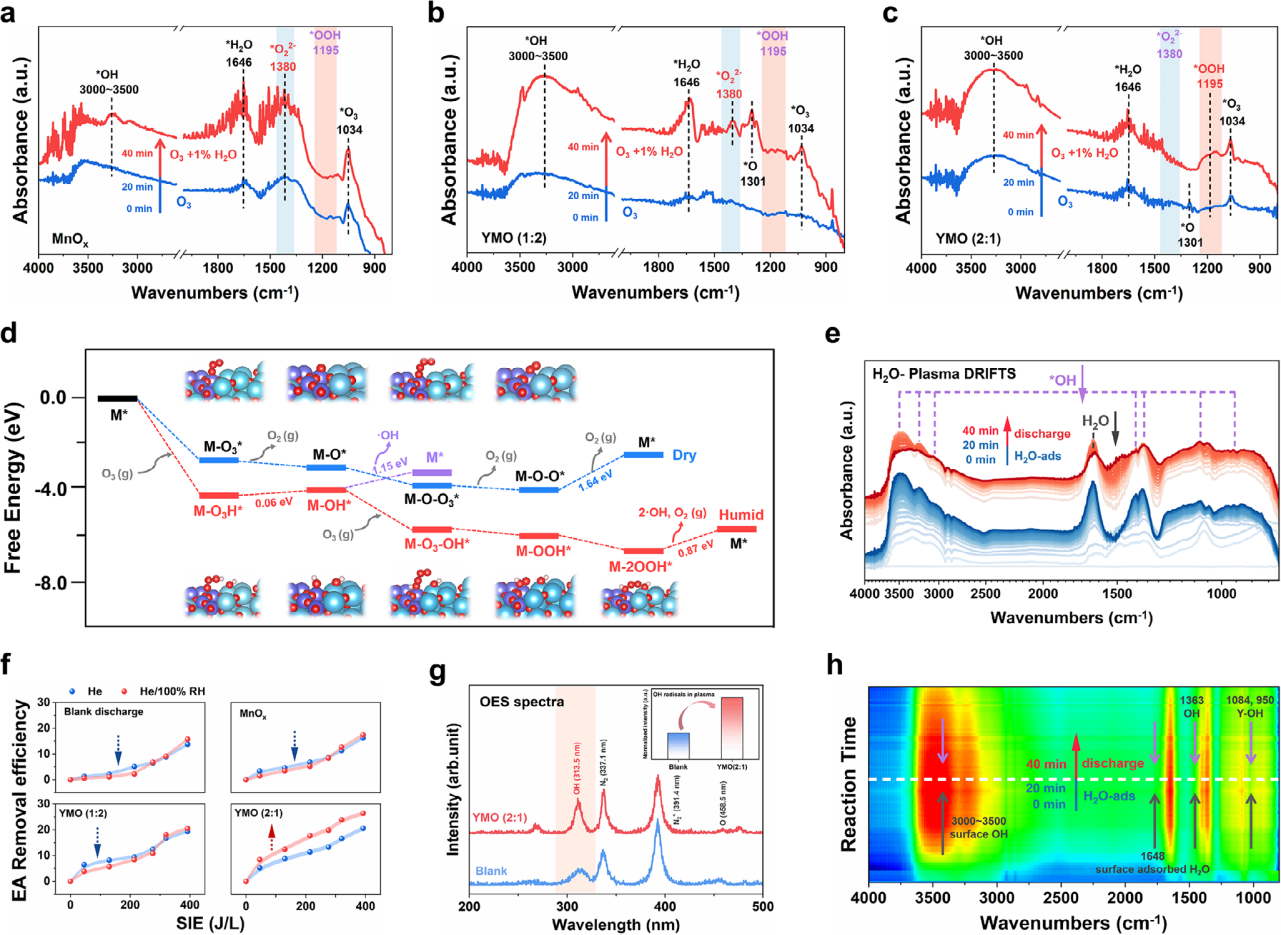
In situ DRIFTS of O_3_ decomposition process on the surface of a) MnO_x_, b) YMO (1:2), and c) YMO (2:1) in dry and humid conditions. d) Calculated Gibbs free energy of O_3_ decomposition on YMO (2:1) under humid conditions, where the asterisk (*) represents the active sites. e) In situ plasma DRIFTS spectra of YMO (1:2) in H_2_O/He and h) its Contour map. f) EA removal efficiency when discharging at He or He/100% RH for different samples. g) Optical emission spectrum for DBD plasma reactor with/without catalysts packing at He/100% RH, and the inset in Figure 4g: the normalized intensity of peak attributed to OH radical.

We first prepared Y_x_Mn_y_O_x+2y_ catalysts with different molar ratios of Y and Mn via the sol‐gel method (Figure 1b). From X‐ray diffraction (XRD) patterns (Figure S1a,b, Supporting Information), pure MnO_x_ is well assigned to the tetragonal Mn_3_O_4_ (JCPDS No. 24–0734), exhibiting the aggregation of nanoparticles with (211) crystal plane exposing in scanning electron microscopy (SEM) and transmission electron microscopy (TEM) images (Figure 2d‐1,d‐4,2d‐5). With the increasing amount of Y atoms, characteristic peaks in XRD attributed to Y‐related metallic oxides appear. XRD Rietveld refinement indicates YMO (1:4) is a composite of YMn_2_O_5_ (62.68%) and Mn_3_O_4_ (37.32%) (Figures S1c and S2a, the refined lattice parameters are shown in Table S1, Supporting Information), while YMO (1:2) and YMO (1:1) are the pure YMn_2_O_5_ with (121) crystal plane exposure (**Figure** **2**a,d‐7) and pure YMnO_3_ with (112) crystal plane exposure (Figures S1d and S2b, Supporting Information), respectively. YMO (2:1) and YMO (4:1) are the composite of YMnO_3_ and Y_2_O_3_ with different proportions (Figure 2b, Figure S1e, Supporting Information), in which YMnO_3_ (112) and Y_2_O_3_ (222) nanoparticles tightly touch each other (Figure 2d‐9). Apparently, by means of adjusting the additional amount of Y atom (Figure 2c), the crystal structure of the original MnO_x_ is modulated to new ternary mullite oxides or perovskites like YMn_2_O_5_ or YMnO_3_, as well as the composite oxides like YMn_2_O_5_/Mn_3_O_4_ or YMnO_3_/Y_2_O_3_.

Further observing in SEM, samples containing Y atoms are all loose and porous structures contributed by the citric acid thermolysis during preparation (Figure 2d‐2,1d‐3, Figure S2, Supporting Information). Benefiting from this, the specific surface areas increase from 4.05 m^2^ g^−1^ (MnO_x_) to more than 22 m^2^ g^−1^ (all Y‐related samples) in BET results (Figure S3, Supporting Information), facilitating the adsorption of EA molecules. The X‐ray photoelectron spectroscopy spectra verify the successful construction of Y_x_Mn_y_O_x+2y_ composites again (XPS, Figure S4, Supporting Information). Notably, the O 1s spectrum exhibits that the content of O_c_ (531.9 eV, attributed to structural H_2_O or OH groups)^[^ ^20^ ^]^ keeps greatly elevating along with increasing Y atom, implying that the Y─Mn─O oxides greatly promote the adsorption of water vapor. H_2_O‐temperature programmed desorption (H_2_O‐TPD, Figure 2e; Figure S5, Supporting Information) further verified that both the physical‐adsorbed H_2_O (desorption peaks at 100≈200 °C) and chemical‐adsorbed H_2_O (400≈600 °C)^[^ ^21^ ^]^ are sharply increased by increasing Y content thanks to its special water vapor affinity. That is, under a humid environment, water vapor can be quickly captured and adsorbed onto those catalysts surface especially with higher Y content.

The specific H_2_O adsorption site and its influence on O_3_ adsorption were then investigated via the density functional theory (DFT) calculations (Figure 2f). It is observed that both O_3_ and H_2_O would adsorb on Mn atom of MnO_x_, implying their severe competitive adsorption, and leading to insufficient active sites for O_3_ adsorption. While for Y‐containing samples (YMO (1:2) and YMO (2:1)), a new Y site is specifically provided for the adsorption of H_2_O molecule, thus avoiding the occupy of Mn sites and keeping the well adsorption of O_3_ even under humidity. In detail, the adsorption energy of O_3_ molecule on YMO (1:2) (−3.68 eV) is more negative than that of pure MnO_x_ (−2.54 eV) and YMO (2:1) (−3.18 eV), indicating that O_3_ is more easily to adsorb and be activated on YMO (1:2). While H_2_O molecule prefers to adsorb on the surface of YMO (2:1) catalyst with the special interface of YMnO_3_‐Y_2_O_3_ (E_ads_ = −1.89 eV). When O_3_ and H_2_O co‐existing, the adsorption energy for all is much more negative than that of single adsorption especially on YMO (2:1) (E_ads_ = −4.57 eV), implying that the co‐adsorption of O_3_ and H_2_O is exothermic and thermodynamically favorable. Particularly, a hydrogen bond between the H atom of the adsorbed H_2_O and the O atom of the adsorbed O_3_ is formed on the surface of YMO (2:1), enhancing the interaction between the two species and facilitating their cooperative decomposition. In a word, dual sites of Mn and Y site for O_3_ and H_2_O adsorption, respectively, are successfully constructed in Y_x_Mn_y_O_x+2y_, potentially contributing to transforming H_2_O to active ·OH.

### 2.2 Co‐Decomposition of O_3_ and H_2_O to Form ·OH in Plasma

After anchoring on the surface of catalysts, the adsorbed H_2_O is prone to be activated and dissociated by capturing electrons from materials. From Bader charge calculation, H_2_O can obtain 0.049, 0.073, and 0.15 e^−^ on the surface of MnO_x_, YMO (1:2) and YMO (2:1), respectively (Figure S6, Supporting Information), indicating that H_2_O molecule prefers to be activated on YMO (2:1). This is contributed by the Y^3+^ site and the special charge transfer channel in YMO (2:1). As shown in XPS Y 3d spectra in Figure S4c (Supporting Information), Y^2+^ is the primary in YMO (1:2) or other low‐Y‐containing samples, while highly enhanced content of Y^3+^ is obtained in YMO (2:1) due to the existence of Y_2_O_3_. Y^3+^ ([Kr]4d^0^5s^0^) possessing vacant orbitals for electron capture is considered as the typical Lewis acid site,^[^ ^22^ ^]^ as demonstrated in Pyridine‐IR spectra (**Figure** **3**a), Y_2_O_3_ and YMO (2:1) show a much higher amount of total L‐sites (the Pyridine adsorption at 50 °C) and strong L‐sites (200 °C).^[^ ^23^ ^]^ The strong L‐sites (Y^3+^) can mediate the interfacial hydrogen bond reconfiguration, thus enhancing the interfacial water to capture electrons and proton transfer on the catalyst surface,^[^ ^24^ ^]^ facilitating its activation and dissociation. Meanwhile, there is a strong covalent interaction between Y atoms of Y_2_O_3_ and O atoms of YMnO_3_ as verified by the Electronic location function (ELF, Figure S7, Supporting Information). Then, charge difference distribution (DIFF, Figure 3b) declares that intense electrons accumulate at the interface and transfer quickly from YMnO_3_ to Y_2_O_3_, and then transfer to the adsorbed H_2_O accelerating its dissociation. The dissociation process of adsorbed H_2_O was then calculated in Figure 3c. The energy release of 0.93 eV is observed during the formation of *OH on the surface of YMO (2:1), while additional energy expenditure of 0.84 and 0.41 eV are required on MnO_x_ and YMO (1:2). This suggests that introducing Y^3+^ site is favorable for the water dissociation to generate surface *OH, as with XPS results. The formation of *OH is also evidenced through FTIR spectroscopy that a new peak located at 1084 cm^−1^ attributed to Y─OH^[^ ^25^ ^]^ vibration appears for all Y‐cotaining samples (Figure S8 and Table S2, Supporting Information), along with the highly enhanced intensity of O─H stretching (3376 cm^−1^)^[^ ^26^ ^]^ especially for samples with higher content of Y, compared with pure MnO_x_. That is, the YMO (2:1) sample with fast charge transfer and a strong Lewis acid site (Y^3+^) realizes the dissociation of adsorbed H_2_O into *OH, which provides a key role for the formation of ·OH during O_3_ decomposition.

As widely reported, the performance of O_3_ decomposition is directly decided by the redox property of the samples, which were evaluated by H_2_‐temperature programmed reduction (H_2_‐TPR) and O_2_‐temperature programmed desorption (O_2_‐TPD) (described in Figure S9, Supporting Information). It is observed the comprehensive redox ability is first increasing and then decreasing along with the increasing Y content, and YMO (1:2) exhibits the best redox ability thanks to its excellent reducibility and greatest oxygen adsorption and mobility. The active radical intermediates derived from O_3_ decomposition under dry and humid conditions were then analyzed by electron paramagnetic resonance (EPR, Figure 3d‐2i, other samples see in Figure S10, Supporting Information). When exposed to dry O_3_ conditions, YMO (1:2) possesses the strongest DMPO‐·O_2_^−^ and DMPO‐·^1^O_2_ signal than that of other samples thanks to its excellent redox ability. While for DMPO‐·OH, the signal intensity increases with the content of Y, and YMO (2:1) performs the best due to its abundant surface H_2_O/OH species and passable redox ability. When introducing water vapor, different phenomena are observed. The intensity of ·O_2_^−^ and ·^1^O_2_ are reducing for samples with lower Y content especially on YMO (1:2), implying the severe water‐poisoning behavior. The intensity of all three radicals increases with increased Y content, and YMO (2:1) exhibits the strongest signals, which is at a comparable level compared with that in dry conditions. Notably, the intensity of ·OH is even stronger than that in dry conditions. This declared that H_2_O molecules do participate in the O_3_ decomposition reactions, introducing a poison effect at low Y content while benefits at high Y content.

For an in‐depth understanding of the co‐decomposition behavior of O_3_ and H_2_O, in situ, diffused reflectance infrared Fourier transform spectroscopy‌ (in situ DRIFTS) was developed (**Figure** **4**a–c). Samples are exposed to dry and humid O_3_ airflow (O_3_+1% H_2_O) in sequence. The peaks at 3000–3500 and 1646 cm^−1^ are associated with the stretching vibration of hydroxyl groups and bending vibration of H_2_O,^[^ ^25^, ^26^ ^]^ respectively. Apparently, the intensity of peaks at 1034 cm^−1^ belonging to physically adsorbed O_3_ molecules^[^ ^27^ ^]^ on the three samples under humid are stronger than that under dry O_3_ airflow, implying that the adsorbed H_2_O/*OH can facilitate O_3_ adsorption, in good consistent with the DFT calculations in Figure 2f. Significantly, the band at 1380 cm^−1^ ascribed to peroxide (O_2_^2−^),^[^ ^6b^ ^]^ the key intermediates for O_3_ decomposition,^[^ ^28^ ^]^ is observed on MnO_x_ under both of dry and humid conditions (Figure 4a). This declares its accumulation and hard to dissociate from active sites, hindering the subsequent reactions. Thus, MnO_x_ exhibits poor O_3_ decomposition and ROS generation as mentioned. For samples of YMO (1:2), no *O_2_^2−^ is detected after O_3_ exposure in dry conditions (Figure 4b), demonstrating again its fast desorption and excellent O_3_ decomposition ability. After adding water vapor, the *O_2_^2−^ and *O (1301 cm^−1^) appear, which indicates that the O_3_ depletion process is severely slowed down due to the adsorption of H_2_O, as with EPR results. Differently, slight *O_2_^2−^ and *O are observed on the surface of YMO (2:1) under dry O_3_ condition (Figure 4c), which totally disappears after H_2_O inletting, along with the appearance of a new peak at 1195 cm^−1^ attributed to *OOH.^[^ ^29^ ^]^ This suggests that the introduction of H_2_O molecules changes the O_3_ decomposition pathway and diminishes the *O_2_^2−^ accumulation on YMO (2:1), thus contributing to the enhanced generation of ROS especially ·OH.

DFT calculations were further performed to study the possible changed pathways. On the surface of YMO (2:1) (Figure 4d), under dry conditions, O_3_ follows a routine decomposition pathway of forming *O and *O_2_^2−^ as with literature, and the speed‐control step is the desorption of *O_2_^2−^ requiring energy expenditure of 1.64 eV. This key energy barrier can decrease to 0.97 eV on YMO (1:2) (Figure S11, Supporting Information), resulting in its excellent O_3_ decomposition performance as proved again. Under humid conditions, an interaction is formed between the first adsorbed O_3_ and the neighboring adsorbed H_2_O, forming *O_3_H. O_3_ and H_2_O would co‐dissociate to produce two *OH intermediates, along with the desorption of O_2_ molecule. This step requires additional energy expenditures of 0.06 eV. Thereafter, direct desorption of *OH to form ·OH needs 1.15 eV energy expenditure (the purple line), which may only occur with the assistance of plasma and will be discussed later. It is thermodynamically favorable that another O_3_ molecule reacts with *OH to generate *O_3_─OH intermediates, which can quickly decompose to *OOH and a desorbed O_2_ molecule, as detected in DRIFTS in Figure 4c. Similarly, identical reactions occur on the second *OH site and form two *OOH intermediates. Finally, two *OOH decompose to generate two ·OH radicals and one O_2_ molecule, and the catalytic cycle is over. This step is the rate‐determining step with an energy requirement of 0.87 eV, which is much lower than the energy barrier under dry conditions (1.64 eV as mentioned). On the surface of YMO (1:2), however, the adsorbed H_2_O is hard to dissociate to form *OH as mentioned, thus O_3_ prefers to follow the traditional decomposition pathway (Figure S11, Supporting Information). Influenced by the adsorbed H_2_O molecule, additional energy of 0.54 and 2.62 eV are required for the formation of *O─O_3_ intermediate and the desorption of *O_2_^2−^, respectively, claiming it is thermodynamically unfavorable. Thus, large amounts of *O_2_^2−^ and *O accumulate on the catalysts’ surface in Figure 4b. In brief, the activation ability of O_3_ and H_2_O of the sample are co‐adjusted by adjusting the ratio of Y and Mn, and the two processes are out of synchrony. YMO (2:1) sample gets the greatest balance, and a new OH‐accompanied pathway with much lower energy barriers for O_3_ decomposition is achieved on YMO (2:1), transforming the adsorbed H_2_O to active ·OH by virtue of this process, as well as ensuring total decomposition of O_3_ under humid condition.

Furthermore, the directly converting H_2_O/*OH to ·OH radicals by the plasma itself is also taken into consideration, except for the decomposition of plasma‐generated O_3_. Control tests of discharging at pure He or He/100% RH were conducted to avoid the possible influence of O_2_ or O_3_ (Figure 4f). It shows that water vapor would hinder EA removal for blank samples due to its severe quenching effect on high‐energy electrons (in pure He plasma, EA is degraded only from cracking reactions collided by high‐energy electrons). On samples of MnO_x_ and YMO (1:2), inhibition is also observed especially at lower inputting energy, possibly owing to their poor ability to trap and dissociate of H_2_O, and most H_2_O molecules are still floating in the discharge atmosphere. While on the surface of YMO (2:1), the existence of H_2_O highly improves EA removal efficiency, suggesting that the surface adsorbed H_2_O/*OH may be easier to convert to free ·OH radicals and apply to oxidation reactions. In situ, DRIFTS was developed to trace the conversion of H_2_O in plasma (Figure 4e,h). Upon the injection of water vapor, the intensity of characteristic peaks for the surface hydroxyl group (3000≈3500, 1363, 1084, and 950 cm^−1^) and the surface adsorbed H_2_O molecule (1648 cm^−1^) gradually increase, indicating again the adsorption and partial dissociation of H_2_O molecules. Subsequently, stop H_2_O introduction and turn on the plasma discharge, the intensity of all the bands gradually decreases, implying their dissociation. The gaseous radicals during plasma discharge in humid conditions were then detected by the Optical emission spectrum (OES, Figure 4g), which directly observes that the intensity of the band at 313.5 nm ascribed to ·OH radicals^[^ ^30^ ^]^ can be highly enhanced when packing YMO (2:1). That is, the surface adsorbed H_2_O/*OH can easily transform to active ·OH radicals thanks to the attack of e* in plasma. Apparently, this process will be in favor of the mentioned OH‐accompanied O_3_ decomposition pathway, accelerating the desorption of *OH and *OOH originally requiring considerable energies as mentioned. In a word, water vapor can be successfully converted to active ·OH on the surface of YMO (2:1) with the assistance of O_3_ and e* in plasma, along with the generation of a considerable amount of ·O_2_^−^ and ·^1^O_2_.

### 2.3 Performance of Plasma‐Catalytic Degradation of Ethyl Acetate

**Figure 5:**
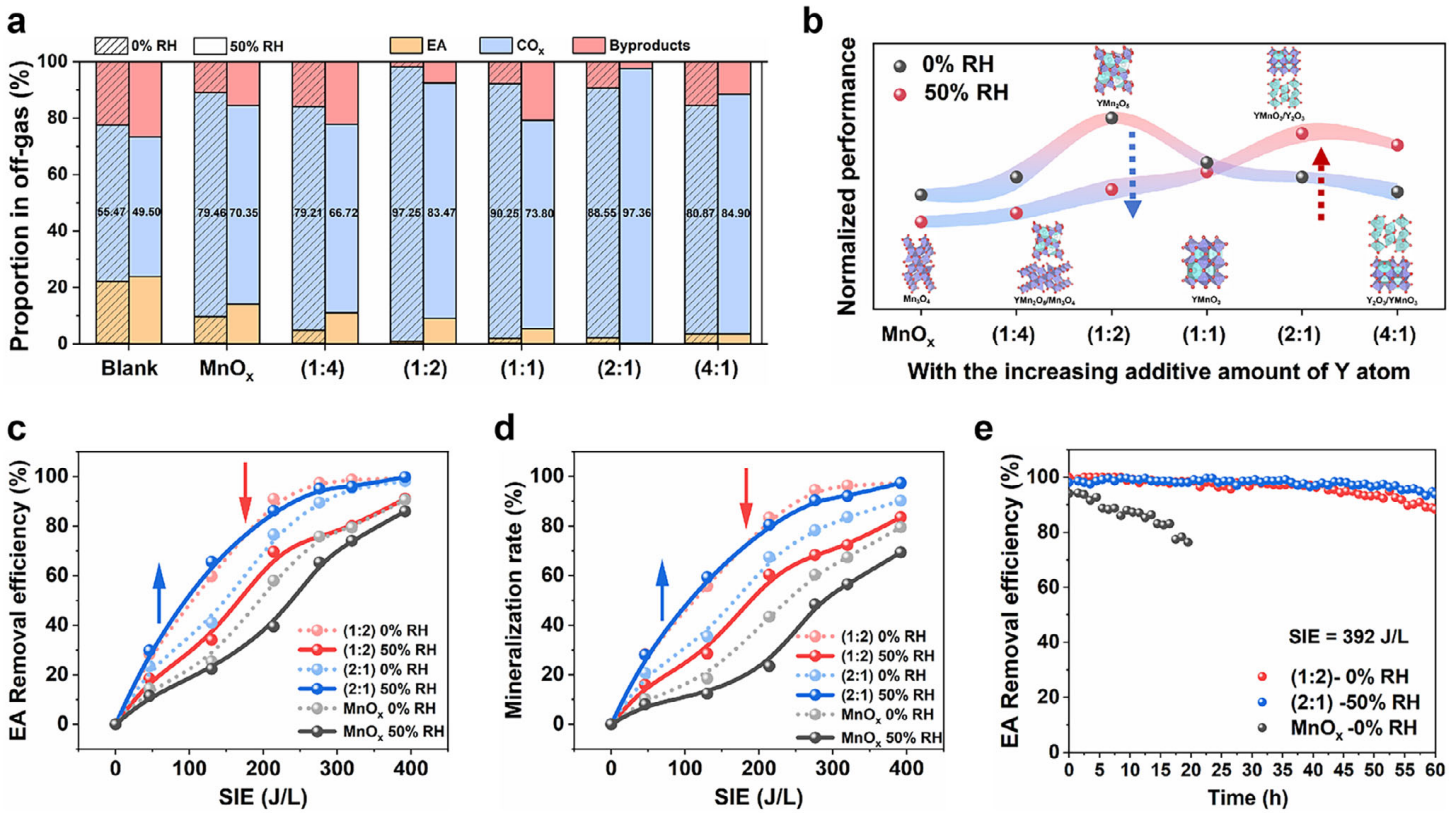
a) The proportion of different carbon in the exhaust for samples at SIE of 392 J L^−1^. b) The normalized performance of the as‐prepared samples with the increasing amount of Y atom under 0% and 50% RH. c) EA removal efficiency and d) mineralization rate for pure MnO_x_, YMO (1:2), and YMO (2:1) under 0% RH and 50% RH. e) Long‐time tests under SIE of 392 J L^−1^.

To assess the effect of this H_2_O‐utilizing strategy on the performance of plasma‐catalysis system, the degradation of ethyl acetate by plasma‐catalysis was conducted in a packed‐bed DBD reactor under dry or humid conditions (the experimental system seen in Figure S12, Supporting Information, the characteristic diagram of the power discharge seen in Figure S13, Supporting Information). As shown in Figure 5a,b, and Figure S14 (Supporting Information), a small part of EA can be removed with an extremely poor mineralization rate in the blank sample, by the reactions of EA with e* and ·O in plasma.^[^ ^31^ ^]^ However, this provides a way to pre‐active the EA molecule, accelerating its following degradation on the surface catalysts. After catalysts packing, under dry conditions (0% RH), the activity first increases and then decreases along with the increasing amount of Y atom, and YMO (1:2) performs the best, along with the least amount of escape O_3_ in the outlet (Figure S15, Supporting Information). EA removal efficiency of YMO (1:2) is 59.74% at only 130 J L^−1^, and reaches 99.28% at 392 J L^−1^ (Figure 5c, red dashed line). This is much higher than that of pure MnO_x_ (25.36% at 130 J L^−1^, and 90.61% at 392 J L^−1^).

The mineralization rate improves from 12.53% (MnO_x_) to 55.74% (YMO (1:2)) at 130 J L^−1^, reaching a maximum of 97.25% at 392 J L^−1^ (Figure 5d). Its O_3_ utilization rate is calculated to be ≈100% thanks to its lowest *O_2_^2−^ desorption energy as discussed (Figure S16, Supporting Information). Physical contributions are excluded as discussed in Figures S17 and S18 (Supporting Information). Control experiments of changing the system from one‐stage to two‐stage directly determine the critical contributions of the degradation and utilization of plasma‐generated O_3_ to EA degradation (Detailed analysis seen in Figures S19 and S20, Supporting Information). Excellent stability is also observed for YMO (1:2) under dry conditions (Figure 5e), and a marked change in its structure is not observed after tests (Figure S21, Supporting Information).

Significantly, the variation trend of the performance of the as‐prepared samples changes after adding water vapor (Figure 5a,b). Evident inhibiting effects are introduced for samples with lower Y content, while surprising promotions are achieved for higher‐Y‐content samples. Particularly, a sharp decrease in EA removal efficiency (34.08% at 130 J L^−1^, and 91.01% at 392 J L^−1^) and mineralization rate (28.46% at 130 J L^−1^, and 83.47% at 392 J L^−1^) on YMO (1:2) is observed at 50% RH (Figure 5c,d, red solid line), along with poor O_3_ utilization rate of only 59.82%. This phenomenon is due to the highly enhanced energy barrier for *O_2_^2−^ desorption in the existence of undissociated H_2_O molecules, resulting in the steep decline of ROS generation, as noted. The higher the water content, the worse the performance (Figure S22a, Supporting Information).

On the contrary, the EA removal efficiency of YMO (2:1) enhanced from 41.04% to 65.62% at 130 J L^−1^, and reached 99.78% at 392 J L^−1^, when changing the relative humidity from 0% RH to 50% RH (Figure 5c, blue line). Its mineralization rate also improves from 35.47% to 59.35% at 130 J L^−1^, and from 90.25% to 97.36% at 392 J L^−1^ (Figure 5d). During this process, the O_3_ utilization rate can be well maintained and even higher than that under dry conditions, lifting from 89.95% to 96.82% (Figure S16, Supporting Information). This is totally contributed by the abundant generation of ROS especially ·OH on the surface of YMO (2:1) via the artful OH‐accompanied O_3_ decomposition pathway as discussed before. In addition, the excellent performance remains well between 30% RH to 90% RH (Figure S22b, Supporting Information), and exhibits remarkable long‐term stability without obvious structure change (Figure 5e, Figure S23, Supporting Information). In brief, this special H_2_O‐utilizing strategy can not only settle the traditional catalyst poison problem induced by H_2_O, but also enhance the performance of plasma‐catalytic degrading VOCs under humid conditions, transforming the traditional poison effects of water vapor into great benefits.

### 2.4 Critical Intermediates and the Reaction Pathways

**Figure 6:**
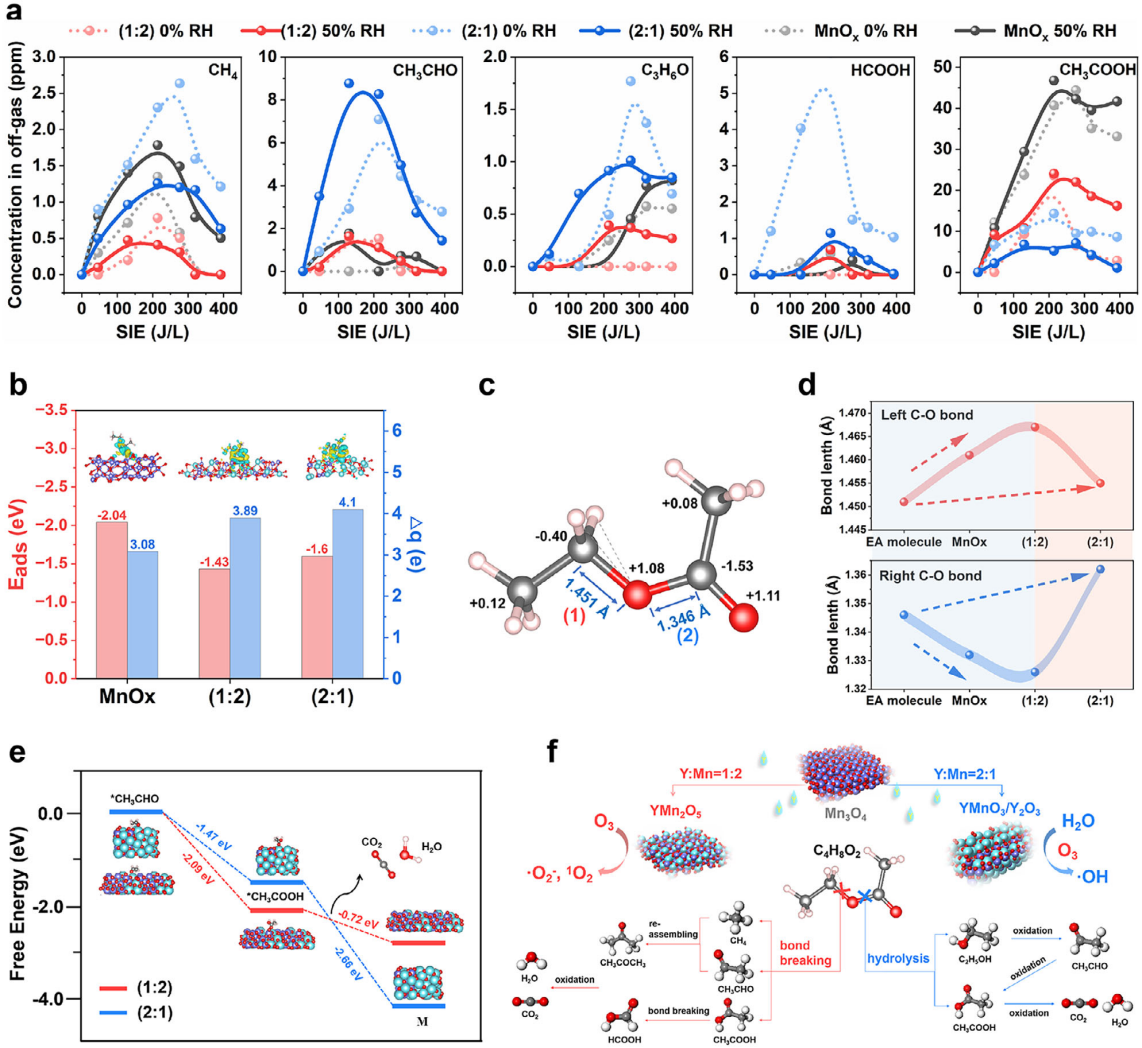
a) Quantitative analysis of CH_4_, CH_3_CHO, C_3_H_6_O, HCOOH, and CH_3_COOH under different conditions. b) The adsorbed energy (Eads) of EA on MnO_x_, YMO (1:2), and YMO (2:1) and the calculated Bader charge (△q), and the inset is the DIFF between EA and the samples (the isosurface set to 0.0012 eV Å^−3^). c) The electron distribution and C─O bond length of the original EA molecule. d) The length evolution of the C─O bond of EA when adsorbed on different samples. e) Calculated Gibbs free energy of the oxidation of CH_3_CHO and CH_3_COOH. f) Possible two pathways of EA molecule on YMO (1:2) and YMO (2:1).

The possible by‐products during reactions were analyzed by GC‐MS (Figure S24 and Table S3, Supporting Information), and the dynamic concentration of the five primary intermediate by‐products (CH_4_, CH_3_CHO, C_3_H_6_O, HCOOH, and CH_3_COOH) were traced during the whole process (Figure 6a). Overall, similar variation tendencies of all by‐products are observed for YMO (1:2) and pure MnO_x_, which mainly work through the traditional O_3_‐decomposition pathway as discussed previously. CH_3_COOH is the primary intermediate with a concentration up to 20–40 ppm in off‐gas especially under humid conditions. While for YMO (2:1), working primarily through the new pathway of co‐decomposition of O_3_ and H_2_O, the distribution of by‐products and their dynamic tendency are totally different, and CH_3_CHO is the key intermediate. Notably, the formation of the intermediates is greatly regulated by water vapor, where the concentration of CH_3_COOH can be greatly controlled and even below 5 ppm under humid conditions. This suggests again that the existence of water vapor on YMO (2:1) surface promotes EA degradation and mineralization.

Detailed DFT calculations were developed to understand the origin of the different distributions of the by‐products for the two types of samples. Figure S25 (Supporting Information) exhibits the adsorption of EA molecules on different surfaces. Pure MnO_x_ exhibits the lowest E_ads_ (−2.04 eV), implying that EA is the easiest to adsorb on it (Figure 6b). However, adsorption alone without activation is not enough. DIFF studies the local electronic interaction between EA and catalysts, and the detail Bader charge (△q) is calculated to be 3.08 e^−^ on MnO_x_, much smaller than that on YMO (1:2) (△q = 3.89 e^−^) and YMO (2:1) (△q = 4.10 e^−^), respectively. This suggests that EA prefers to be activated on the surface of YMO (1:2) and YMO (2:1), contributing to further oxidation reactions. Then, special attentions are paid to the local electron distribution and the bond length of the C─O bond in adsorbed EA molecule, since the C─O bond has been reported to possess the lowest dissociation energy and easy to be attack during reactions.^[^ ^32^ ^]^ When comparing with the initial status of EA molecule (Figure 6c), it is found that the left C─O bond (labeled (1) in Figure 6c) is elongated to 1.461 Å and 1.467 Å, respectively, when EA adsorbing on MnO_x_ and YMO (1:2) (Figure 6d; Figure S25, Supporting Information), while the right C─O bond (labeled (2)) is shortened. This illustrates that the left C─O bond of EA prefers to break under the attack of O_3_, following the pathway of bond breaking. Under this path, CH_3_COOH would be generated in large quantities as shown in Figure 6a. While the opposite phenomenon is observed for YMO (2:1) the right C─O bond is elongated to 1.362 Å and barely changes in the left C─O bond, suggesting that the right C─O bond would be broken in priority and the hydrolysis process plays the leading role under this condition. Accordingly, C_2_H_5_OH and CH_3_COOH are the products during the hydrolysis process, and C_2_H_5_OH would be quickly oxidized to CH_3_CHO. Thus, hardly any C_2_H_5_OH is detected in off‐gas while CH_3_CHO is produced in large amounts.

Specifically, CH_3_CHO and CH_3_COOH would both be produced under the two pathways, but the dominant by‐product is different as mentioned. Gibbs free energy calculations were then developed to understand the conversion process of the two primary by‐products, as displayed in Figure 6e. It is found that 1.47 eV of energy would be released during the oxidation of *CH_3_CHO to *CH_3_COOH on YMO (2:1), which can be greatly promoted on the surface of YMO (1:2) with 2.09 eV of energy release. This indicates that the adsorbed *CH_3_CHO is easier to oxidize to *CH_3_COOH on YMO (1:2), and thus the un‐reacted *CH_3_CHO would desorb on YMO (2:1) and emit. Furthermore, the oxidation of *CH_3_COOH to CO_2_ and H_2_O releases 0.72 and 2.66 eV of energy on the surface of YMO (1:2) and YMO (2:1), respectively, implying that YMO (2:1) is the superior platform for the oxidation of *CH_3_COOH, and the other would be in favor of its desorption. Thus, a considerable amount of CH_3_COOH is detected in off‐gas for YMO (1:2), as discussed before.

In brief, as displayed in Figure 6f, YMO (1:2) with the best redox ability can totally decompose the plasma‐generated O_3_ to active ROS (·O_2_^−^, ·^1^O_2_) following the traditional pathway under try conditions. Under the attack of those ROS, the left C─O bond in EA first breaks, and CH_3_COOH serves as the primary by‐product due to its large energy requirement for further oxidation. Differently, a new pathway of co‐decomposition of O_3_ and H_2_O to form ·OH is constructed on the surface of YMO (2:1) thanks to its outstanding capacity of H_2_O anchoring and dissociating. EA is hydrolyzed on this catalyst surface (the right C─O bond first broken) with the help of the surface *OH group and CH_3_CHO is the critical intermediate due to the poor oxidation ability of the catalyst to CH_3_CHO.

### 2.5 Extension to the Degradation of Other Typical VOCs

Additionally, the universality of this proposed H_2_O‐utilizing strategy was investigated on the plasma‐catalytic degradation of other typical VOCs (Toluene, Acetone, P‐xylene, and the mixture VOCs containing four VOCs). When adding water vapor, no catalyst poisoning phenomena but highly enhanced VOCs degradation performance are observed even for the mixture VOCs (Figure S26, Supporting Information), thanks to the co‐dissociation of O_3_ and H_2_O on the surface of YMO (2:1) as previously proved, generating abundant active ·OH. Specifically, for chain‐VOCs like EA and acetone, both of the removal and the mineralization rate can be promoted. While for aromatic hydrocarbon‐VOCs such as toluene and p‐xylene, this strategy primarily facilitates the VOCs mineralization, implying the great contributions of ·OH to the ring opening. In a word, this new strategy is universally applicable for VOCs elimination by plasma‐catalysis. Such special H_2_O‐accelerated property artfully addresses the bottlenecks of this technology and realizes its stable operation under real and humid conditions, potentially facilitating its industrialized application.

## 3 Conclusion

This work describes a reverse strategy to transform the traditional poison effect of water vapor into benefits, aided by the self‐generated O_3_ and e* in plasma. We construct Y_x_Mn_y_O_x+2y_ catalysts with dual active sites for the co‐adsorption of O_3_ and H_2_O, where adsorbed H_2_O can be quickly dissociated to *OH groups on YMO (2:1) thanks to the fast interfacial charge transfer and strong Lewis acid sites (Y^3+^). Thereafter, *OH can modulate the O_3_ decomposition behavior and form a new OH‐accompanied pathway with lower energy barriers for O_3_ decomposition under humid conditions, generating active ·OH. The key intermediate species is identified as *OOH determined by in situ DRIFTS and DFT calculations. e* in plasma can promote the desorption of *OH and *OOH during this new course, as well as pre‐active the polluting molecules (EA). Benefiting from the abundant ·OH generation, 99.78% EA degradation and 97.36% mineralization rate are achieved on the surface of YMO (1:2) under a humid environment, with excellent long‐time stability. Transiting the traditional O_3_ decomposition process to the newly H_2_O‐participated process, the first C─O bond‐breaking changes from the left to the right one, and the key intermediate by‐product changes from CH_3_COOH to CH_3_CHO. This H_2_O‐utilizing strategy is also universally applicable for plasma‐catalytic oxidation of other VOCs. This study provides rational design principles for the utilization of water vapor in plasma catalysis, paving a new way for catalysts free from deactivation in applications containing ubiquitous H_2_O.

## 4 Experimental Section

### Synthesis of Y_x_Mn_y_O_x+2y_

Stoichiometric amounts of Y(NO_3_)_3_·6H_2_O, Mn(CH_3_COO)_2_·4H_2_O, and citric acid (1.5 times of molar ratio of the metal salts) were dissolved in 100 mL deionized water. The obtained solution was evaporated and dried at 120 °C, and then calcined at 800 °C for 8 h. Samples with different molar ration of Y and Mn are obtained by adjusting the ratio of precursor salts, and denoted as YMO (x:y) following the molar ratio of Y and Mn (Y:Mn = 1:4, 1:2, 1:1, 2:1, 4:1). For comparison, pure MnO_x_ was prepared by direct calcination of Mn(CH_3_COO)_2_·4H_2_O under the same conditions.

### Characterization

X‐ray diffraction (XRD) patterns of the samples were performed by using MiniFlex600‐C diffractometer with Cu‐Kα (λ = 1.5406 Å) radiation operated at 40 kV and 15 mA. The scanning speed was 10° min^−1^, and the scattering angle was 2 θ. The data collection range is 10°–90°. The Rietveld refinements were performed by using GSAS2 software.

Scanning electron microscopy (SEM, HITACHI UHR, SU 8010) and Transmission electron microscopy (TEM, H‐600, Hitachi, Ltd., Japan) were used to observe the morphology of the samples. The acceleration voltage of the electron microscope in TEM was 100–200 kV. Pretreatment was conducted as following steps, prior to SEM tests: ultrasonic dispersion in anhydrous ethanol, take a small amount of sample on the copper net, drying at 60 °C.

BET (JW‐BK 132F, Beijing) was employed to study the specific surface area and pore structure, respectively. Pretreatment was conducted at 150 °C for 2 h to remove the physically adsorbed impurities. The experimental data were collected below −196 °C.

X‐ray photoelectron spectroscopy (XPS) with a monochromatic Al Kα source (150 W, 1486.6 eV) was used to analyze the surface properties and valence state distribution of the samples, in which the C 1s (284.8 eV) was the reference binding energy.

The temperature‐programmed desorption of O_2_ (O_2_‐TPD), the hydrogen temperature‐programmed reduction (H_2_‐TPR), and the temperature‐programmed desorption of H_2_O (H_2_O‐TPD) were conducted on an Auto Chem II automatic chemical adsorption instrument (BelCata II) equipped with an on‐line mass spectrometer (BelMass). O_2_‐TPD: 0.05 g catalysts were pre‐treated under 5% O_2_/He at 300 °C for 30 min (30 mL min^−1^), and then cooled to room temperature. The catalyst was heated from room temperature to 800 °C at 10 °C min^−1^ in pure He (30 mL min^−1^).

H_2_‐TPR: 0.05 g catalysts were pre‐treated under 5% O_2_/He at 300 °C for 30 min (30 mL min^−1^), and then cooled to room temperature. The catalyst was heated from room temperature to 800 °C at 10 °C min^−1^ in 5% H_2_/He (30 mL min^−1^).

H_2_O‐TPD: 0.05 g catalysts were pre‐treated under He at 400 °C for 30 min (30 mL min^−1^), and then cooled to room temperature. Switch to saturated water vapor to adsorption saturation, and then He flow of 30 mL min^−1^ for 30 min was used to remove the residual water vapor. The catalyst was heated from room temperature to 700 °C at 10 °C min^−1^ in pure He (30 mL min^−1^).

Electron paramagnetic resonance (ESR) measurements were conducted on a Bruker EMX EPR Spectrometer (Bruker A300, Bruker Corp., Billerica, MA). The DMPO (5, 5‐dimethyl‐1‐pyrroline N‐oxide) was adopted as the trapping reagent for free radicals including ·O_2_^−^ and ·OH. The TEMP (2,2,6,6‐ Tetramethylpiperidine) was used as the trapping reagent for ·^1^O_2_. All ESR tests were performed with O_3_ purging at 30 °C under dry conditions of under 1% H_2_O.

### Experimental Device and Catalytic Performance Evaluation

A coaxial packed‐bed dielectric barrier discharge (DBD) reactor was developed in this study, where the metal bar and the metal net act as the high voltage and ground electrode, respectively. The discharge gap and length of the reactor are 3 and 100 mm, respectively. Catalysts (0.2 g) coated onto glass balls (15 g) with a diameter of 2.5 mm are packed in the discharge zone to maintain close exposure between the plasma and catalysts. For comparison, plasma packing with blank glass balls was denoted as “Blank” to exclude the physical packing effect. A modulating pulse power supply (CTP‐2000KP, Suman, China) was employed for the discharge, in which the duty ratio and the modulation frequency were fixed at 60% and 64 Hz, respectively. A high‐voltage probe (P6015A, Tektronix), current probe (CP8030B, Zhiyong), and digital oscilloscope (TDS2012B, Tektronix) were employed to measure the voltage, current, and waveform, respectively. The simulated industrial waste gas (2 L min^−1^, 100 ppm EA, 21 vol. % O_2_ and balanced N_2_, 0%–90% RH) was used as the inlet gases. Other three typical VOCs (toluene, acetone, and p‐xylene) and mixture VOCs (25 ppm ethyl acetate + 25 ppm toluene + 25 ppm acetone + 25 ppm p‐xylene) with inlet concentration of 100 ± 5 ppm are chosen for extended tests under the same conditions. The humidity was supplied by feeding one flow of dry N_2_ through ultrapure water which was contained in a bubbler in a thermostatic bath. All experiments were conducted at ambient room temperature and pressure.

The concentration of all VOCs, CO/CO_2,_ and five specific by‐products (CH_4_, CH_3_CHO, CH_3_COCH_3_, HCOOH, and CH_3_COOH) were measured using an on‐line gas chromatograph (GC, FULI 9790II, China) equipped with a flame ionization detector (FID), an electron capture detector (ECD), and nickel converting equipment. O_3_ was measured with an O_3_ analyzer (UV–100, Eco Sensors in America).

GC‐MS system (GC: Agilent 7890A, MS: Agilent 5975C) equipped with a J&W113‐4332GS‐GasPro chromatographic column (America) was also employed to analyze the components of the off‐gas (containing multiple gaseous products), which was captured by employing an adsorption tube (Tenax TA/Tenax GR) at room temperature for 1 h, and was then released in a thermal desorption instrument (TDI, PERSEE‐TP7, PR China) at 300 °C and fed into the GC‐MS system for analysis.

The specific input energy (SIE), EA removal efficiency (*η*), and mineralization rate (*η*_COx_) were defined and calculated as follows:

(1) $$\begin{array}{ccc} \mathrm{SIE}\;(J/L) & = & P/Q=\mathrm{discharge}\;\mathrm{power}\;(W)\;*\;\\ & & 60\;\left(s/\min\right)\;/\;\mathrm{total}\;\mathrm{flow}\;\mathrm{rate}\;(L\;\min-\mathrm{mathrm}1) \end{array}$$

(2) $$P\;\left(W\right)=\;E\;*\;f\;=\;\mathrm{input}\;\mathrm{energy}\;\left(J\right)\;*\;\mathrm{frequency}\;\left(\mathrm{Hz}\right)$$

(3) $$\begin{array}{ccc} E\;(J) & = & {{\mathrm{smallint}}_{0}}^{T}\;u(t)\;*\;i(t)\mathrm{dt}{=\int_{0}}^{\mathrm{one}\;\mathrm{pulse}\;\mathrm{time}}\\ & & \mathrm{transient}\;\mathrm{voltage}\;(V)\;*\;\mathrm{transient}\;\mathrm{current}\;(A)\mathrm{dt} \end{array}$$

(4) $$\eta \;C_{4}H_{8}O_{2}\;(\%)=\frac{C_{\mathrm{inlet}}\;-\;C_{\mathrm{outlet}}}{C_{\mathrm{inlet}}}*100\%$$

(5) $$\mathrm{Mineralization}\;\mathrm{rate}\;(\%)=\frac{C_{\mathrm{CO}}+C_{\mathrm{CO}2}}{4*C_{\mathrm{inlet}}}\;\;*\;100\%$$

(6) $$\mathrm{Average}\;\mathrm{current}\;(A)=\int_{0}^{T}|i\left(t\right)|\mathrm{dt}/t$$

(7) $$\mathrm{Effective}\;\mathrm{current}\;(A)=\int_{0}^{T}{\left(i\left(t\right)\right)}^{2}\mathrm{dt}/t$$

### In Situ DRIFTS Tests

In situ DRIFTS of O_3_ decomposition: catalysts are pre‐treated at 200 °C for 30 min under N_2_, and then cooled to room temperature. Collect the baseline when it reaches stable. Dry O_3_ (1000 ppm) was first introduced into the reaction cell for 20 min at 30 °C, and then, the inlet gas was switched to the mixed atmosphere of O_3_ and saturated water vapor (1% H_2_O/N_2_) for another 20 min. Signals of in situ DRIFTS on the catalyst surface are collected during the whole process by the infrared detector at 2 min intervals.

In situ plasma DRIFTS: Initially, a predetermined quantity of catalyst was introduced into the reaction cell, followed by purging with He at 150 to remove impurities from the catalyst surface. Subsequently, the catalyst was allowed to cool to room temperature. Water vapor was introduced into the reaction cell for 20 min, and then, stop H_2_O introduction and turn on the plasma discharge for 20 min. Signals of in situ DRIFTS on the catalyst surface are then collected by the infrared detector at 30 s intervals.

### Theoretical Calculations

The density functional theory (DFT) calculations were performed via the “Vienna ab initio simulation package” (VASP 5.4.1). To overcome the deficiency of the standard DFT for Mn atom, the DFT+U method was employed (U = 3.0 eV).^[^ ^33^ ^]^ The cutoff energy and Gaussian smearing width were set to 400 and 0.2 eV, respectively. The K point in the Brillouin zone was set to 3*3*1 within the Monkhorst–Pack grid for both structural optimization and electronic structural calculations. The vacuum slab was set to 15 Å to avoid an interaction error between neighboring supercells. The adsorption energy (E_ads_) was calculated as follows:

(8) $$E_{\mathrm{ads}}=E_{\mathrm{tot}}-(E_{\mathrm{mol}}+E_{\mathrm{material})}$$

where *E*_tot_, *E*_mol,_ and *E*_material_ are the total energy of the adsorption system, the isolated molecule, and the material structure, respectively.

The Gibbs free energies were calculated at 298.15 K, and as defined as:

(9) $$G=E_{\mathrm{DFT}}-\mathrm{TS}+E_{\mathrm{ZPE}}$$

where *E*_DFT_, TS, and *E*_ZPE_ refer to the DFT energy, entropy contribution, and zero‐point energy, respectively.

## Conflict of Interest

The authors declare no conflict of interest.

## Supporting information



Supporting Information

## Data Availability

The data that support the findings of this study are available from the corresponding author upon reasonable request.
