# Supplementary material for: Transforming the Poison Effects of Water Vapor into Benefits Over Adjustable Dual Acid Sites for Stable Plasma‐Catalysis
Source: Adv Sci (Weinh). 2025 Apr 26;12(27):2502123. doi: 10.1002/advs.202502123 (PMC12279242; doi:10.1002/advs.202502123)
Supplement: Supplementary file 1 — Supporting Information [file ADVS-12-2502123-s001.docx]

Supporting Information

**Transforming the Poison Effects of Water Vapor into Benefits over Adjustable Dual Acid Sites for Stable Plasma-Catalysis**

*Si Chen, Sibo Zhang, Lu Fang, Yan Yang^*^, Chenyuan Zhu, Xinyi Dai, Zhengjun Gong^*^, and Fan Dong^*^*

**Table of Contents**

**[1. Supplementary Figures 4](#_Toc7800)**

**[Figure S1](#_Toc3953)**[. (a) XRD pattern of the as-prepared samples. XRD Rietveld refinement of (b) MnOx, (c) YMO (1:4), (d) YMO (1:1), and (e) YMO (4:1). (Yobs: observed Bragg position, Ycal: calculated Bragg position, Yobs-Ycal: difference) 4](#_Toc3953)

**[Figure S2](#_Toc18803)**[. (a-1) SEM and (a2-a3) TEM image for YMO (1:4), (b-1) SEM and (b2-b3) TEM image for YMO (1:1), and (c-1) SEM and (c2-c3) TEM image for YMO (4:1). 4](#_Toc18803)

**[Figure S3](#_Toc14745)**[. BET results of the as-prepared samples. 5](#_Toc14745)

**[Figure S4](#_Toc32728)**[. XPS spectra of the as-prepared samples: (a) O 1s, (b) Mn 2p and (c) Y 3d. 5](#_Toc32728)

**[Figure S5](#_Toc21882)**[. Enlarged view of H](#_Toc21882)_[2](#_Toc21882)_[O-TPD results for MnO](#_Toc21882)_[x](#_Toc21882)_[, YMO (1:4) and YMO (1:2). 6](#_Toc21882)

**[Figure S6](#_Toc22054)**[. The Optimized structures of H](#_Toc22054)_[2](#_Toc22054)_[O molecules adsorbed on (a) MnO](#_Toc22054)_[x](#_Toc22054)_[, (b) YMO (1:2) and (c) YMO (2:1). 6](#_Toc22054)

**[Figure S7](#_Toc8025)**[. (a) Electronic location function (ELF) calculation of YMO (2:1). (b) Project density of states (PDOS) for YMO (2:1). 7](#_Toc8025)

**[Figure S8](#_Toc12878)**[. FTIR results of the as-prepared samples. 7](#_Toc12878)

**[Figure S9](#_Toc32055)**[. H](#_Toc32055)_[2](#_Toc32055)_[-TPR and (g) O](#_Toc32055)_[2](#_Toc32055)_[-TPD of the as prepared samples. 7](#_Toc32055)

**[Figure S10](#_Toc25297)**[. ESR signals of (a) hydroxyl radicals (b) ·](#_Toc25297)^[1](#_Toc25297)^[O](#_Toc25297)_[2](#_Toc25297)_ [and (c) ·O](#_Toc25297)_[2](#_Toc25297)_^[-](#_Toc25297)^ [of different samples with O](#_Toc25297)_[3](#_Toc25297)_ [purging at 30](#_Toc25297) ^[o](#_Toc25297)^[C under dry and humid conditions. 8](#_Toc25297)

**[Figure S11](#_Toc10737)**[. Calculated Gibbs free energy of O](#_Toc10737)_[3](#_Toc10737)_ [decomposition on YMO (1:2) under humid condition, where the asterisk (*) represents the active sites. 9](#_Toc10737)

**[Figure S12](#_Toc19448)**[. Overall principal diagram for the experimental system. 9](#_Toc19448)

**[Figure S13](#_Toc8737)**[. The characteristic diagram of the power discharge: (a) the waveform of the modulating pulse power; (b) the V-I waveform of one square wave; (c) the detail view of the square-wave; (d) the energy of one square-wave. 9](#_Toc8737)

**[Figure S14](#_Toc17308)**[. EA removal efficiency with SIE of the as-prepared samples at (a) 0% RH and (b) 50% RH. 10](#_Toc17308)

**[Figure S15](#_Toc10557)**[. The escape amount of O](#_Toc10557)_[3](#_Toc10557)_ [in the out-let gas during reaction for different samples at (a) 0% RH and (b) 50% RH. 10](#_Toc10557)

**[Figure S16](#_Toc14420)**[. The utilization rate of O](#_Toc14420)_[3](#_Toc14420)_ [for the as-prepared samples under different conditions. 11](#_Toc14420)

**[Figure S17](#_Toc12430)**[. Characteristics of microdischarge for various packing catalysts in plasma at SIE of 392 J/L: (a) packing with blank glass balls, (b) MnO](#_Toc12430)_[x](#_Toc12430)_[, (c) YMO (1:2), (d) YMO (2:1). 11](#_Toc12430)

**[Figure S18](#_Toc26382)**[. The variation of average current during the variation of input voltage from 6.0 to 8.5 KV under the conditions of plasma cooperating with different catalysts. 11](#_Toc26382)

**[Figure S19](#_Toc30449)**[. The scheme of catalysts placed in plasma zone and downstream of the plasma. 12](#_Toc30449)

**[Figure S20](#_Toc14931)**[. Performance of (a-b) YMO (1:2) and (c-d) YMO (2:1) placed in plasma zone and downstream of the plasma under the relative humidity of 0% and 50%. 13](#_Toc14931)

**[Figure S21](#_Toc12156)**[. XRD patterns for YMO (1:2) before and after long-time experiment. 14](#_Toc12156)

**[Figure S22](#_Toc2248)**[. EA removal efficiency with SIE of (a) YMO (1:2) and (b) YMO (2:1) sample under the relative humidity of 0~90%. 14](#_Toc2248)

**[Figure S23](#_Toc11904)**[. XRD patterns for YMO (2:1) before and after long-time experiment. 15](#_Toc11904)

**[Figure S24](#_Toc21679)**[. The gas phase by-products detected by GC-MS. 15](#_Toc21679)

**[Figure S25](#_Toc22410)**[. The electron distribution and C-O bond length of the EA molecule adsorbed on (a) MnO](#_Toc22410)_[x](#_Toc22410)_[, (b) YMO (1:2) and (c) YMO (2:1). 16](#_Toc22410)

**[Figure S26](#_Toc31859)**[. The degradation of other typical VOCs: the removal efficiency and mineralization of (a, e) toluene (100 ppm), (b, f) acetone (100 ppm), (c, g) p-xylene (100 ppm) and (d, h) mixture VOCs (25 ppm ethyl acetate + 25 ppm toluene + 25 ppm acetone + 25 ppm p-xylene). 16](#_Toc31859)

**[2. Supplementary Tables 17](#_Toc7229)**

**[Table S1](#_Toc3199)**[. The results of XRD Rietveld refinements (performed with GSAS2 software) 17](#_Toc3199)

**[Table S2](#_Toc6983)**[. FTIR band assignments for Figure S8. 18](#_Toc6983)

**[Table S3](#_Toc9463)**[. GC-MS results for the as-prepared samples. 18](#_Toc9463)

**[References 19](#_Toc10176)**

# **Supplementary Figures**


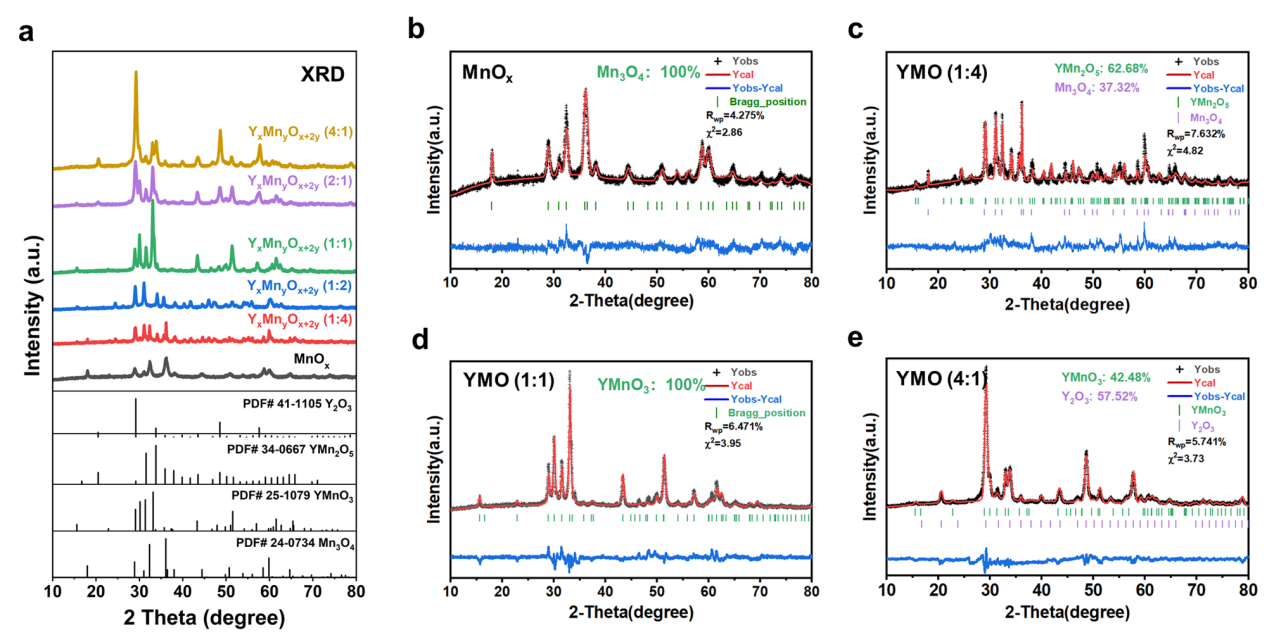


## **Figure S1.** (a) XRD pattern of the as-prepared samples. XRD Rietveld refinement of (b) MnOx, (c) YMO (1:4), (d) YMO (1:1), and (e) YMO (4:1). (Yobs: observed Bragg position, Ycal: calculated Bragg position, Yobs-Ycal: difference)


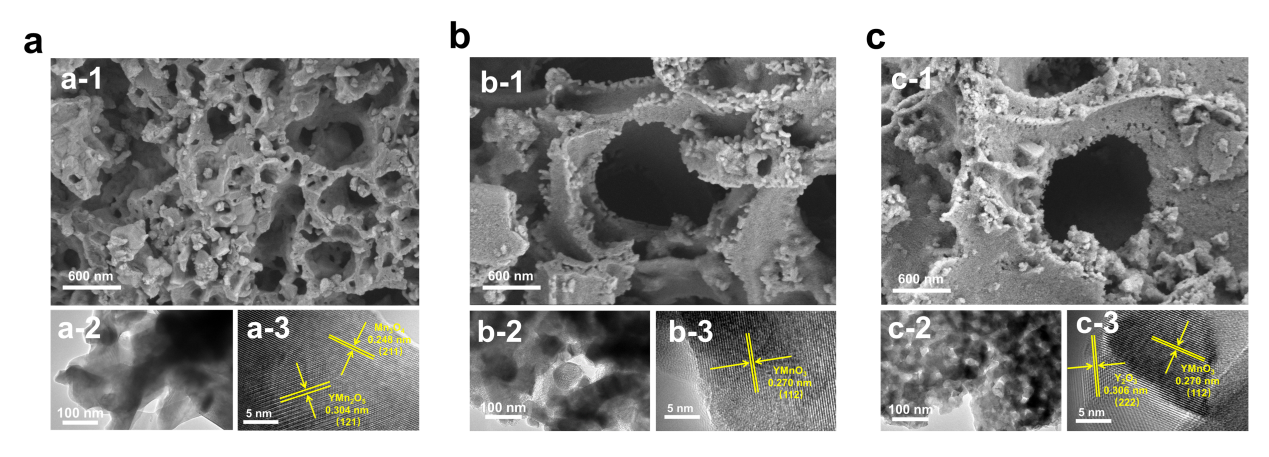


## **Figure S2.** (a-1) SEM and (a2-a3) TEM image for YMO (1:4), (b-1) SEM and (b2-b3) TEM image for YMO (1:1), and (c-1) SEM and (c2-c3) TEM image for YMO (4:1).


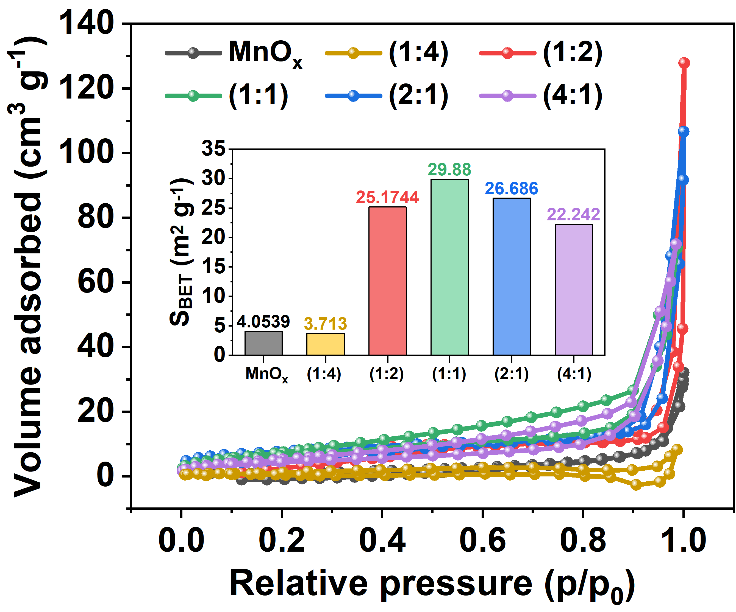


## **Figure S3.** BET results of the as-prepared samples.


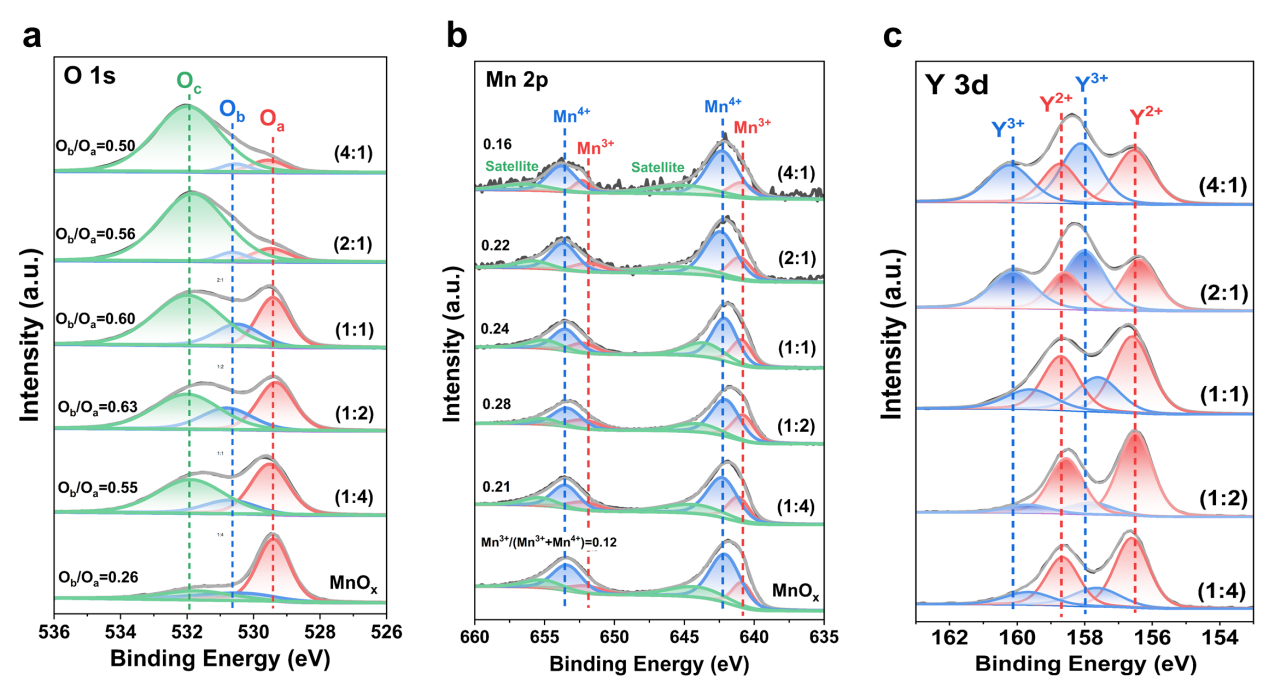


## **Figure S4.** XPS spectra of the as-prepared samples: (a) O 1s, (b) Mn 2p and (c) Y 3d.

The O 1s spectrum can be deconvoluted to three convolution peaks at 529.4, 530.5 and 531.9 eV (**Figure S4a**), ascribed to the lattice oxygen (labeled as Oa), chemisorbed oxygen (Ob) and structural H_2_O or OH groups (Oc), respectively^[2]^. The deconvolution of Mn 2p profile (**Figure S4b**) revealed six peaks assigned to Mn^3+^ (641.4 eV and 652.1 eV), Mn^4+^ (642.8 eV and 654.2 eV) and satellite (645.9 eV and 656.0 eV)^[3]^, respectively. The peaks in the Y 3d region (**Figure S4c**) are also deconvoluted, where the peaks centered at 156.67 eV and 158.6 eV are assigned to Y^2+ [4]^, and peaks at 157.6 eV and 160.0 eV are attributed to Y^3+^.

_
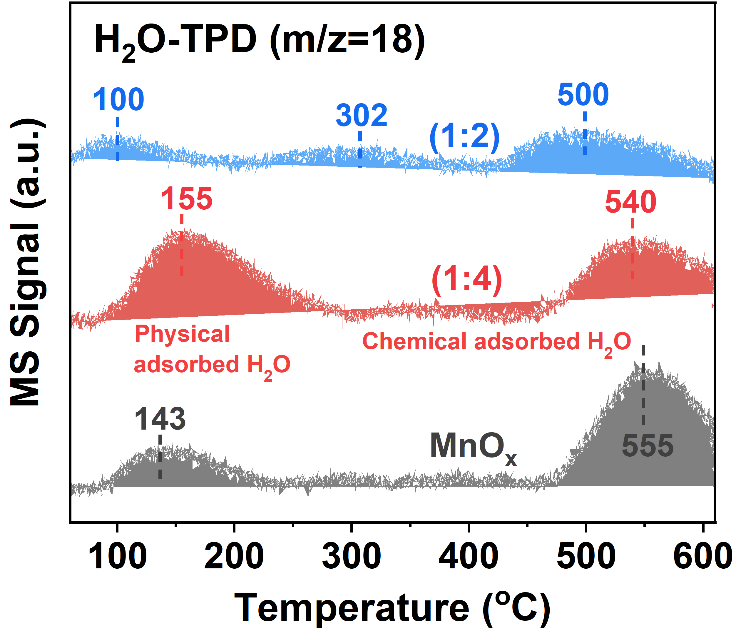
_

## **Figure S5.** Enlarged view of H_2_O-TPD results for MnO_x_, YMO (1:4) and YMO (1:2).


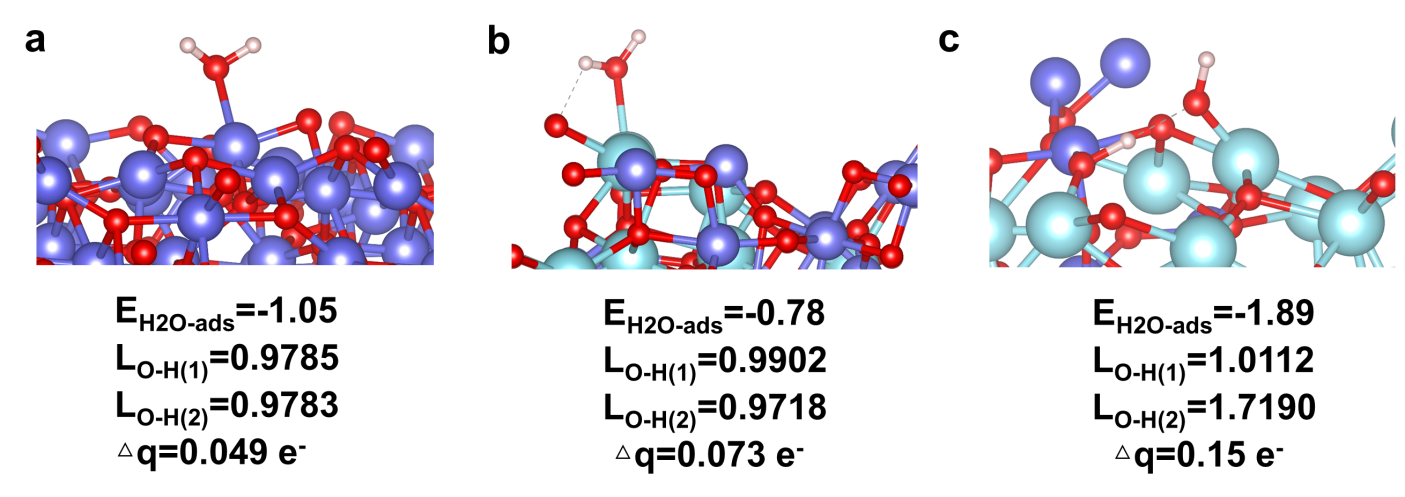


## **Figure S6.** The Optimized structures of H_2_O molecules adsorbed on (a) MnO_x_, (b) YMO (1:2) and (c) YMO (2:1).


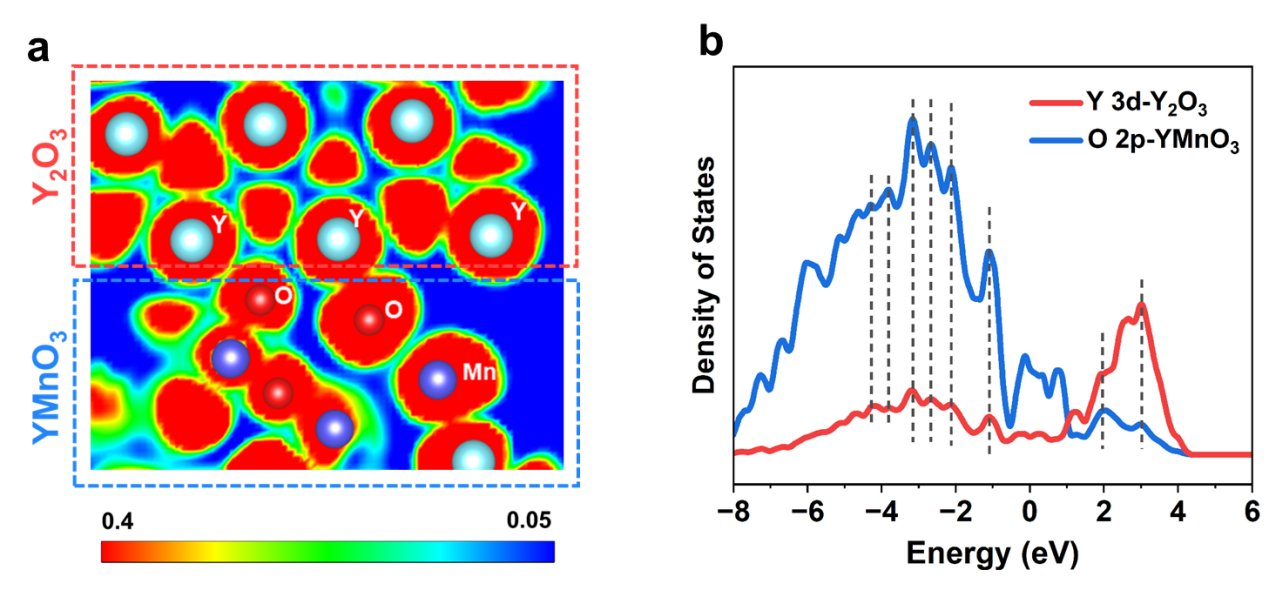


## **Figure S7.** (a) Electronic location function (ELF) calculation of YMO (2:1). (b) Project density of states (PDOS) for YMO (2:1).


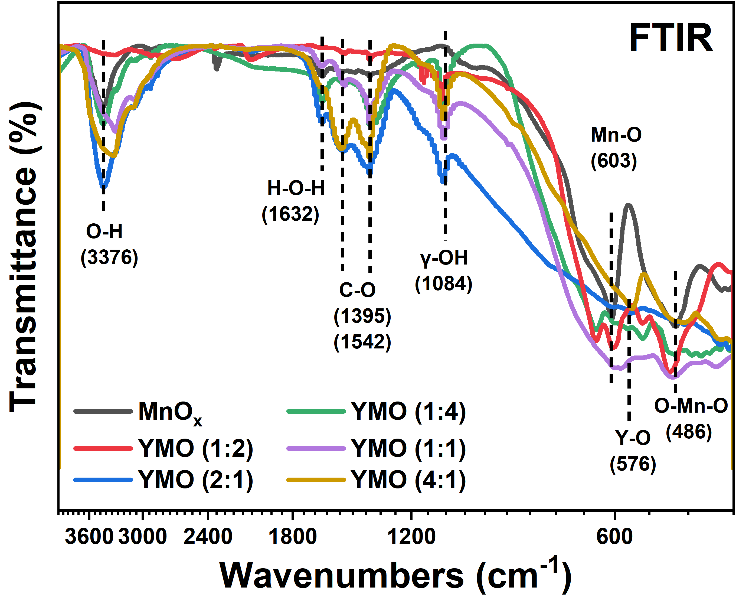


## **Figure S8.** FTIR results of the as-prepared samples.


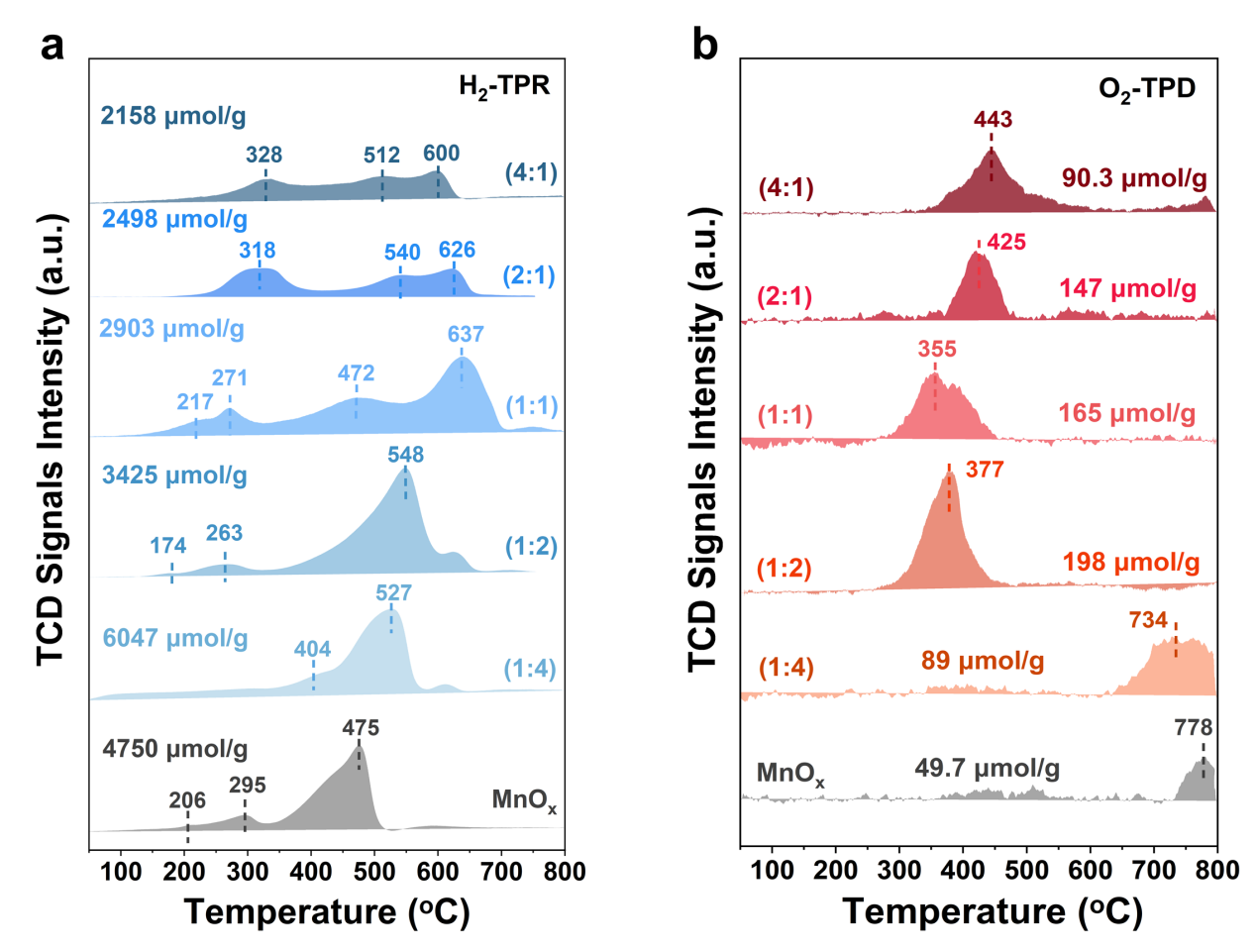


## **Figure S9.** H_2_-TPR and (g) O_2_-TPD of the as prepared samples.

H_2_-TPR profiles (**Figure S9a**) infer that the temperature of the reduction peak of Mn^4+^ to Mn^3+^ (the first desorption peak)^[5]^ exhibits the tendency of increase-decrease-increase with the increasing Y content, along with the firstly increasing and then decreasing H_2_-consumption. It suggests that the total reducibility of samples are decreasing after the introduction of Y species, but the reducibility in low-temperature is slightly lifted. O_2_-TPD (**Figure S9b**) manifests that MnO_x_ and YMO (1:4) display extremely poor oxygen adsorption and oxygen mobility with much higher temperature of desorption peak and lower O_2_ consumption. Further increasing Y content greatly improves the properties, but excessive amount of Y cause slight decline again, and YMO (1:2) is the best. The above results confirm that the redox property is adjusted due to the introduction of Y atom, and YMO (1:2) exhibits the best redox ability thanks to its most content of O_b_, excellent reducibility and greatest oxygen adsorption and mobility.


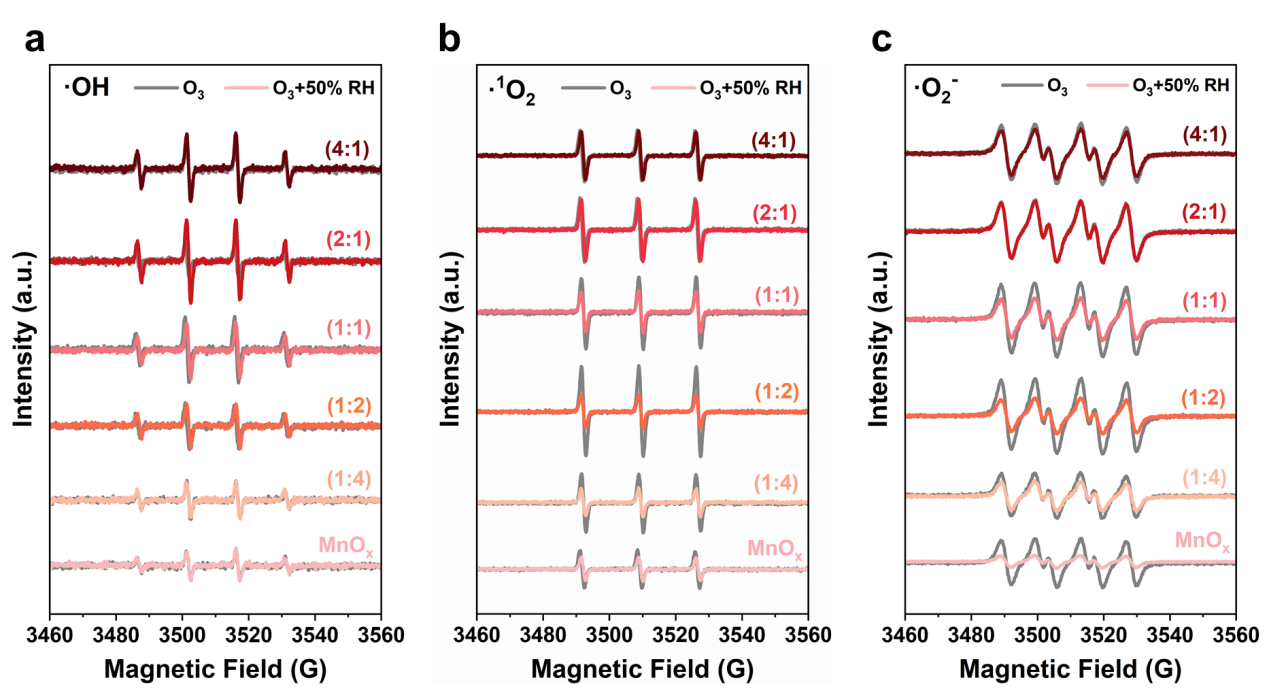


## **Figure S10.** ESR signals of (a) hydroxyl radicals (b) ·^1^O_2_ and (c) ·O_2_^-^ of different samples with O_3_ purging at 30^o^C under dry and humid conditions.


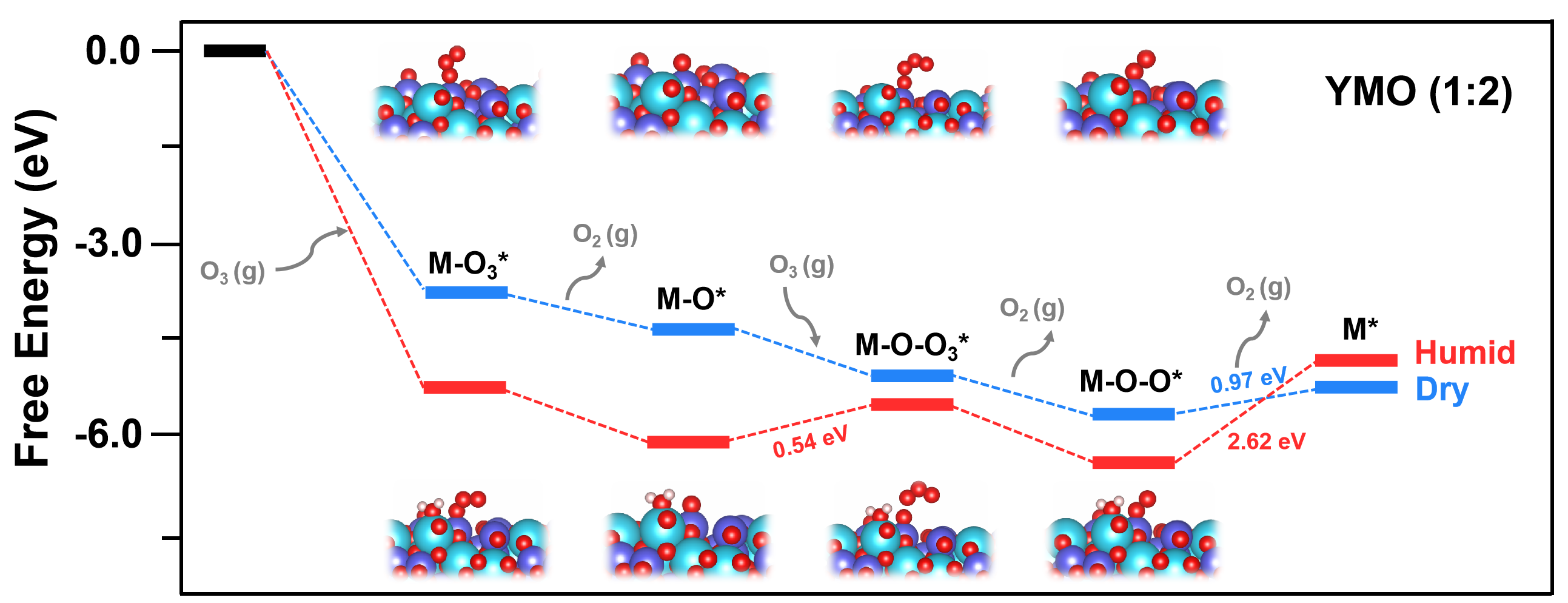


## **Figure S11.** Calculated Gibbs free energy of O_3_ decomposition on YMO (1:2) under humid condition, where the asterisk (*) represents the active sites.


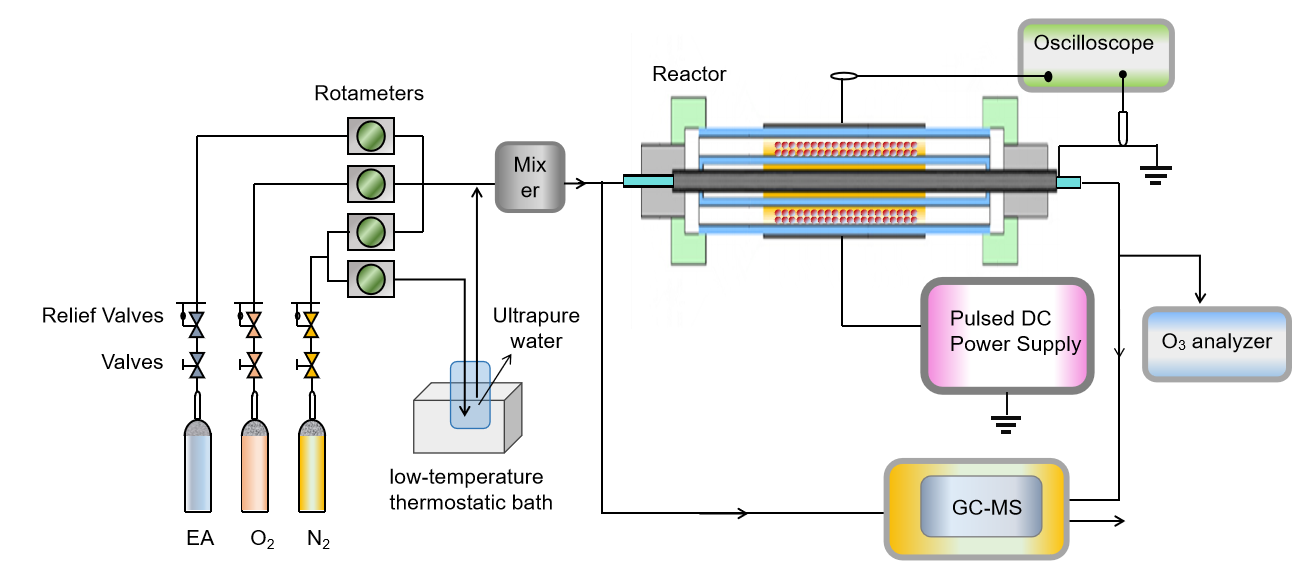


## **Figure S12.** Overall principal diagram for the experimental system.


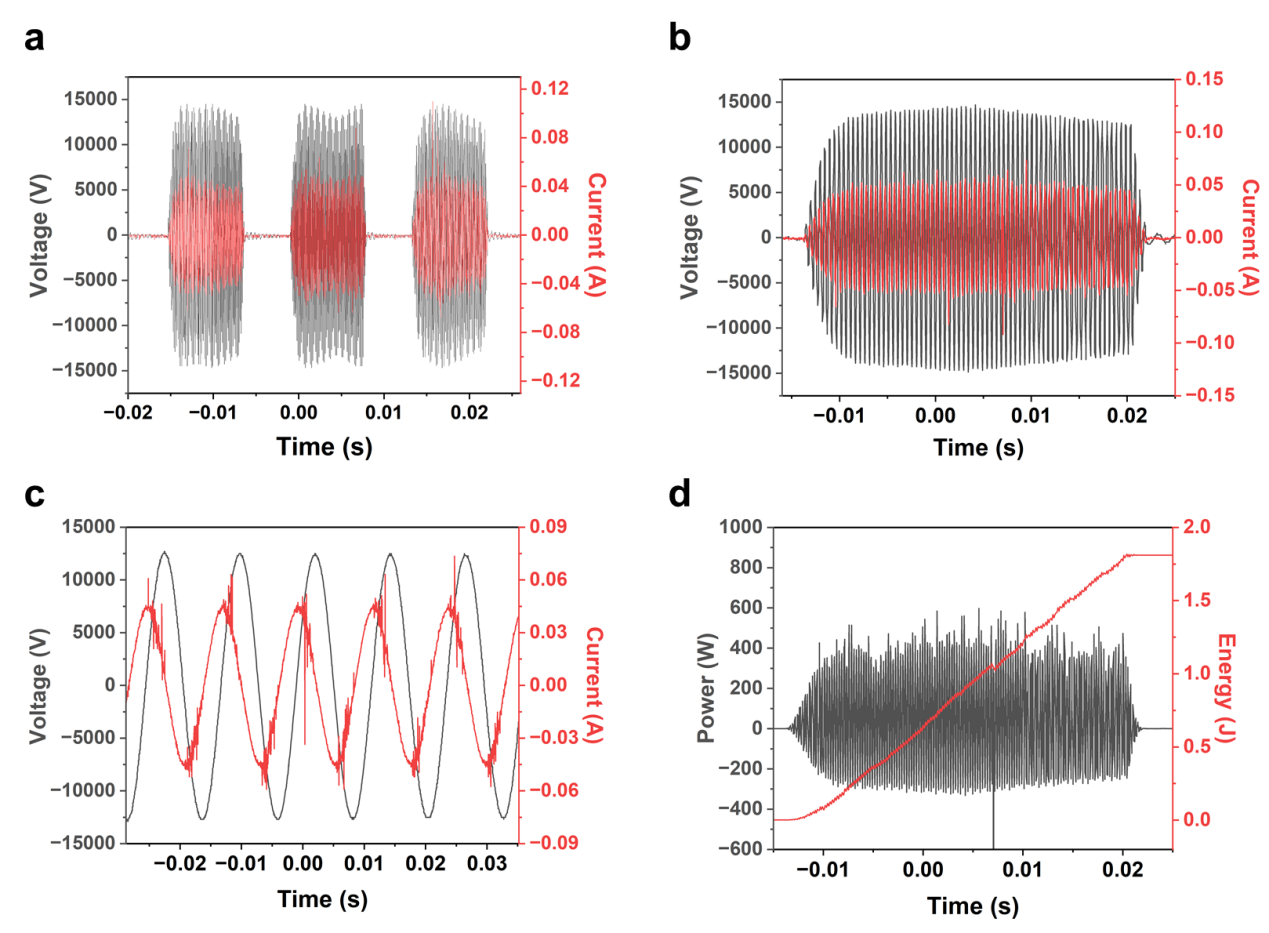


## **Figure S13.** The characteristic diagram of the power discharge: (a) the waveform of the modulating pulse power; (b) the V-I waveform of one square wave; (c) the detail view of the square-wave; (d) the energy of one square-wave.


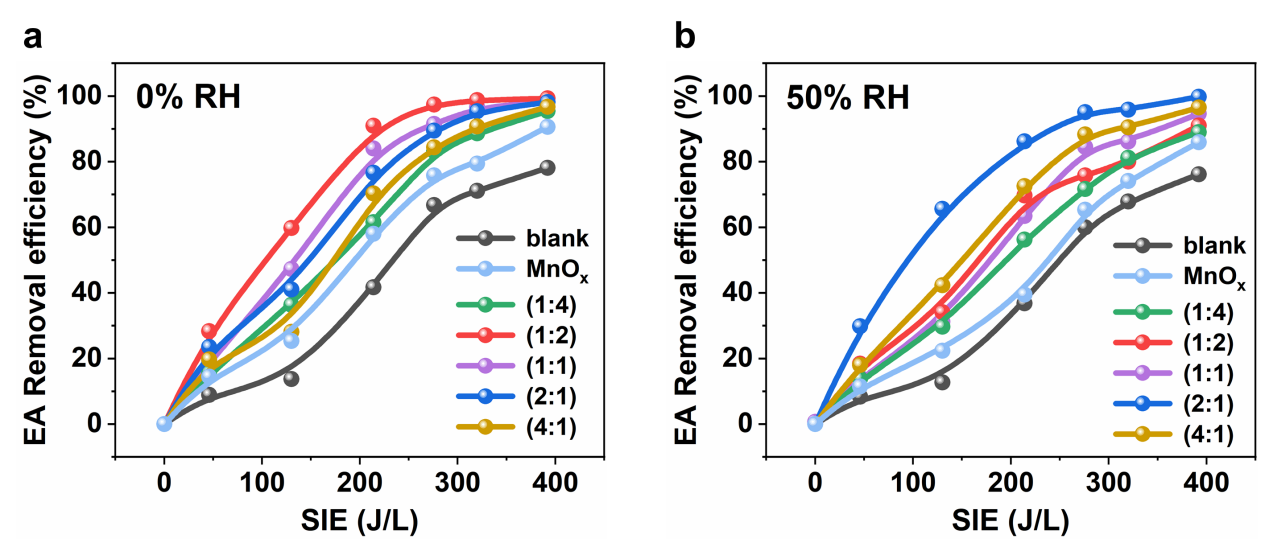


## **Figure S14.** EA removal efficiency with SIE of the as-prepared samples at (a) 0% RH and (b) 50% RH.


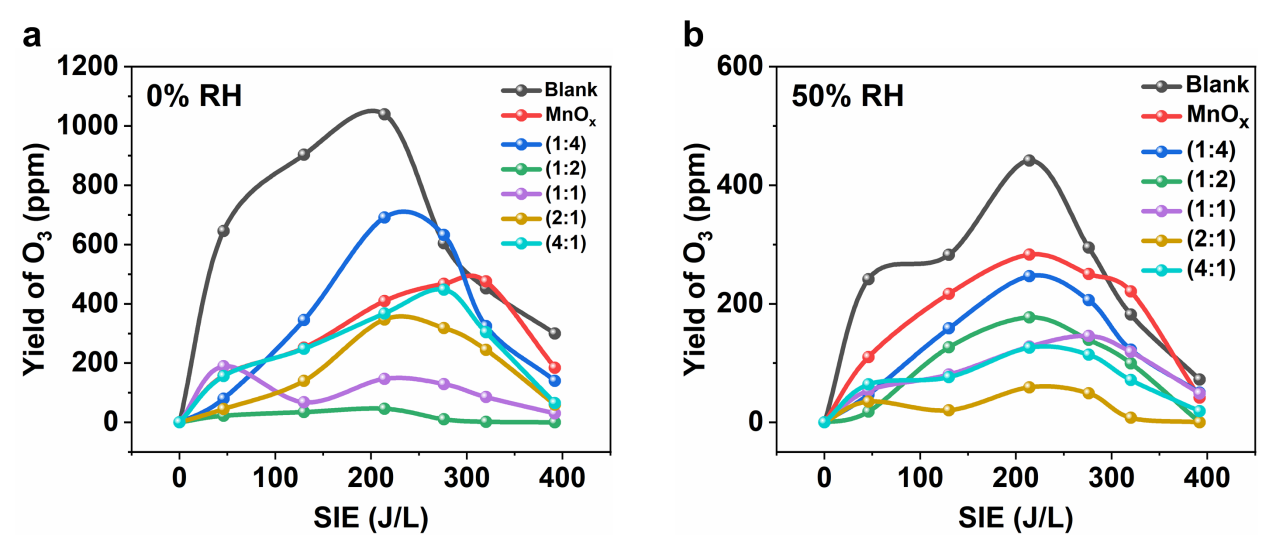


## **Figure S15.** The escape amount of O_3_ in the out-let gas during reaction for different samples at (a) 0% RH and (b) 50% RH.


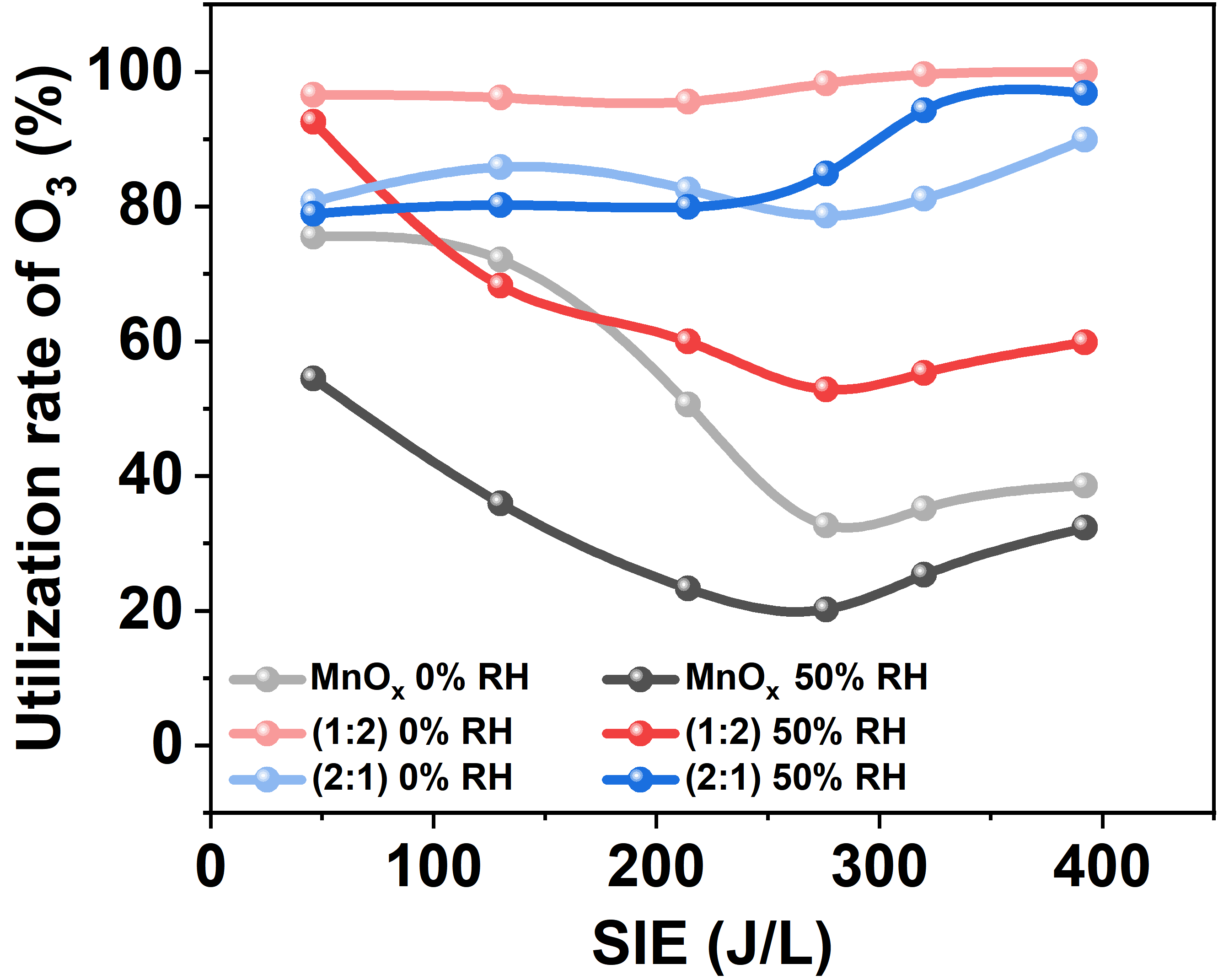


## **Figure S16.** The utilization rate of O_3_ for the as-prepared samples under different conditions.


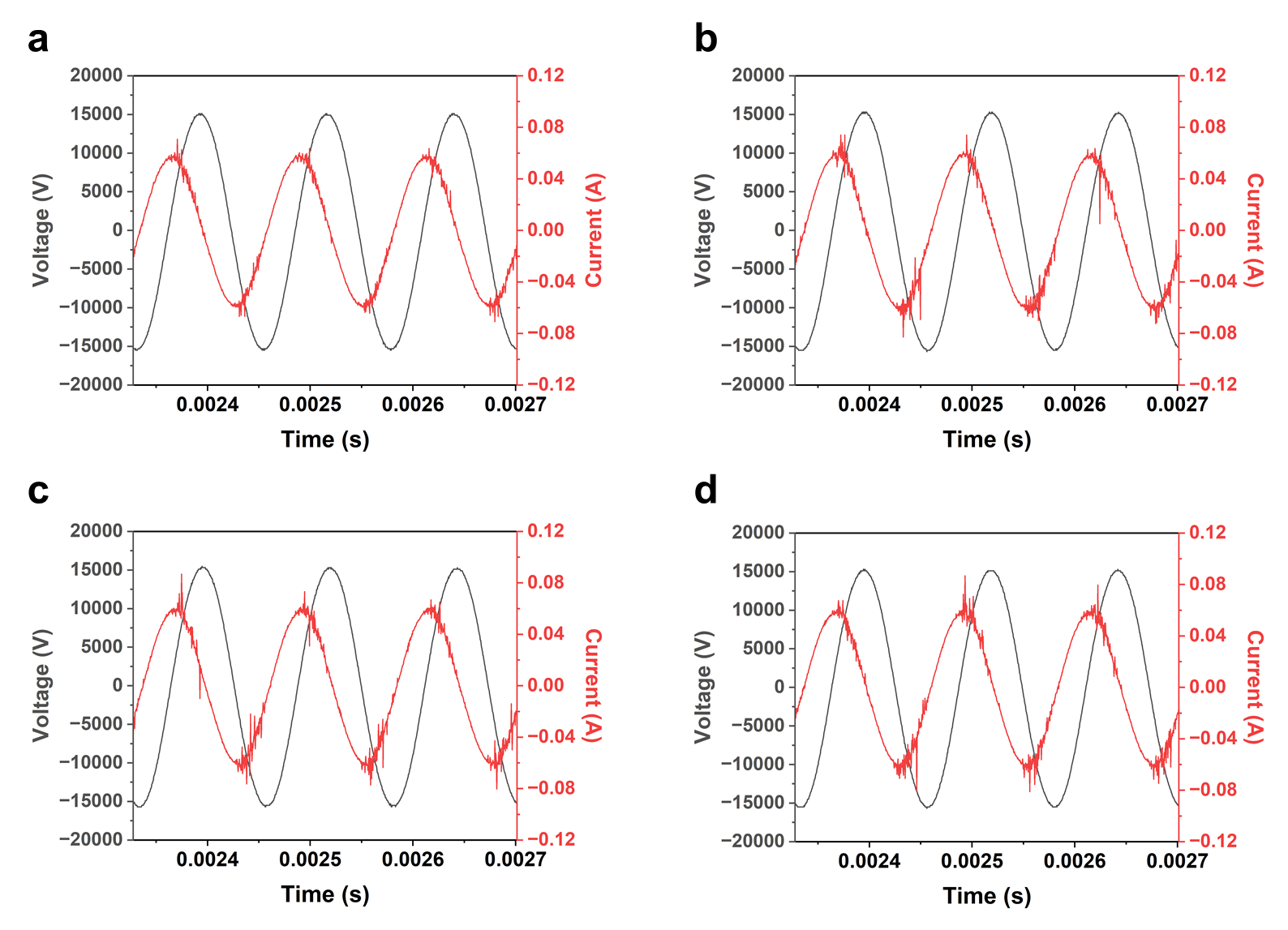


## **Figure S17.** Characteristics of microdischarge for various packing catalysts in plasma at SIE of 392 J/L: (a) packing with blank glass balls, (b) MnO_x_, (c) YMO (1:2), (d) YMO (2:1).


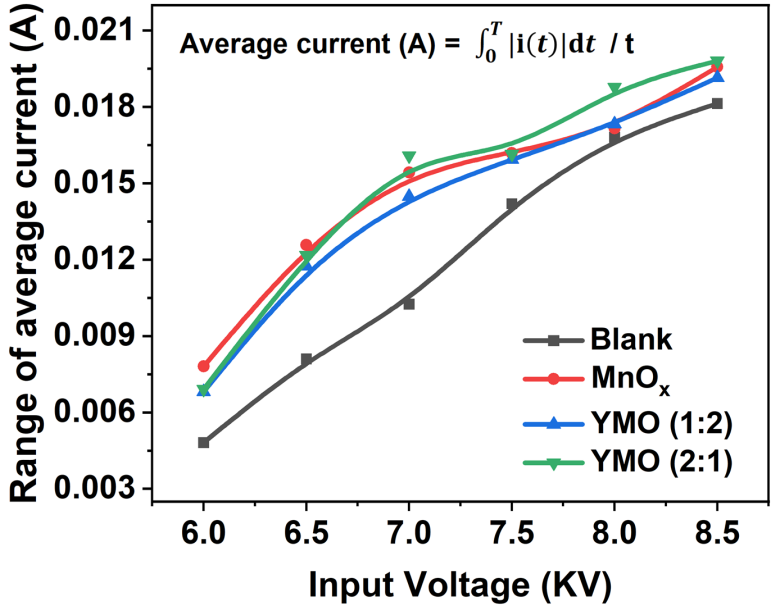


## **Figure S18.** The variation of average current during the variation of the input voltage from 6.0 to 8.5 KV under the conditions of plasma cooperating with different catalysts.

As shown in **Figure S17**, more dense microdischarges are observed after packing catalysts than that of glass balls, owing to the increase of the localized filamentary discharge induced by the presence of catalysts. This could provide abundant “hot” electrons for the initiation of chemical reactions^[6]^. In detail, as displayed in **Figure S18**, similar average current is obtained for YMO (1:2), YMO (2:1) and pure MnO_x_, respectively, determining that the physical effect is not the key for the performance enhancements, other chemical effects are required to investigate deeply.


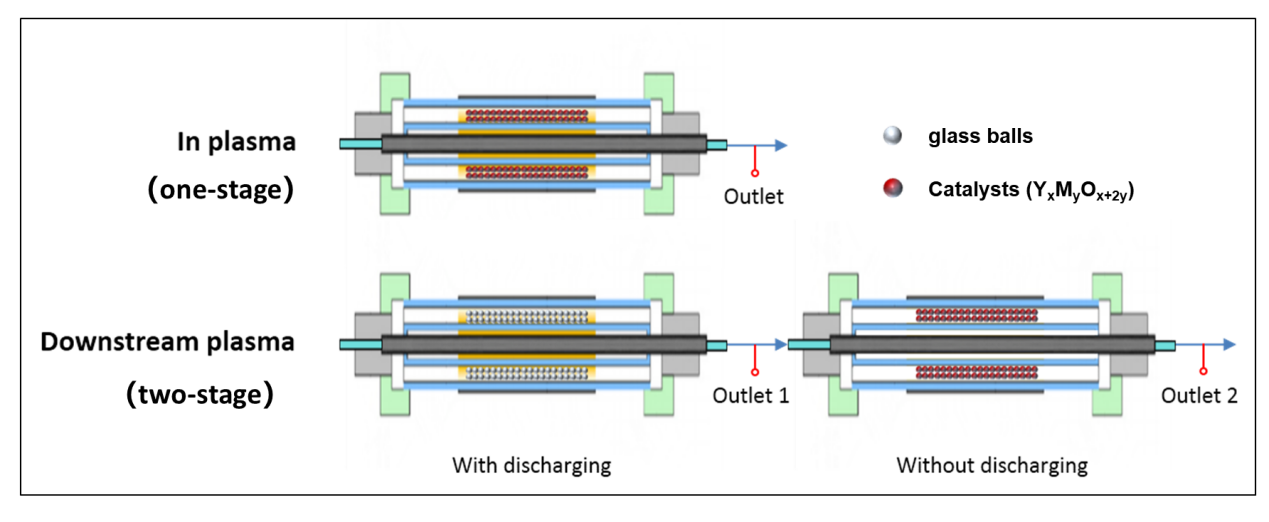


## **Figure S19.** The scheme of catalysts placed in plasma zone and downstream of the plasma.

One-stage: catalysts are placed in the plasma zone, denoted as outlet.

Two-stage: catalysts are placed downstream of the plasma. Gas first go through the plasma zone packed with glass balls, denoted as outlet-1, then flow past the catalyst zone without discharging, denoted as outlet-2.


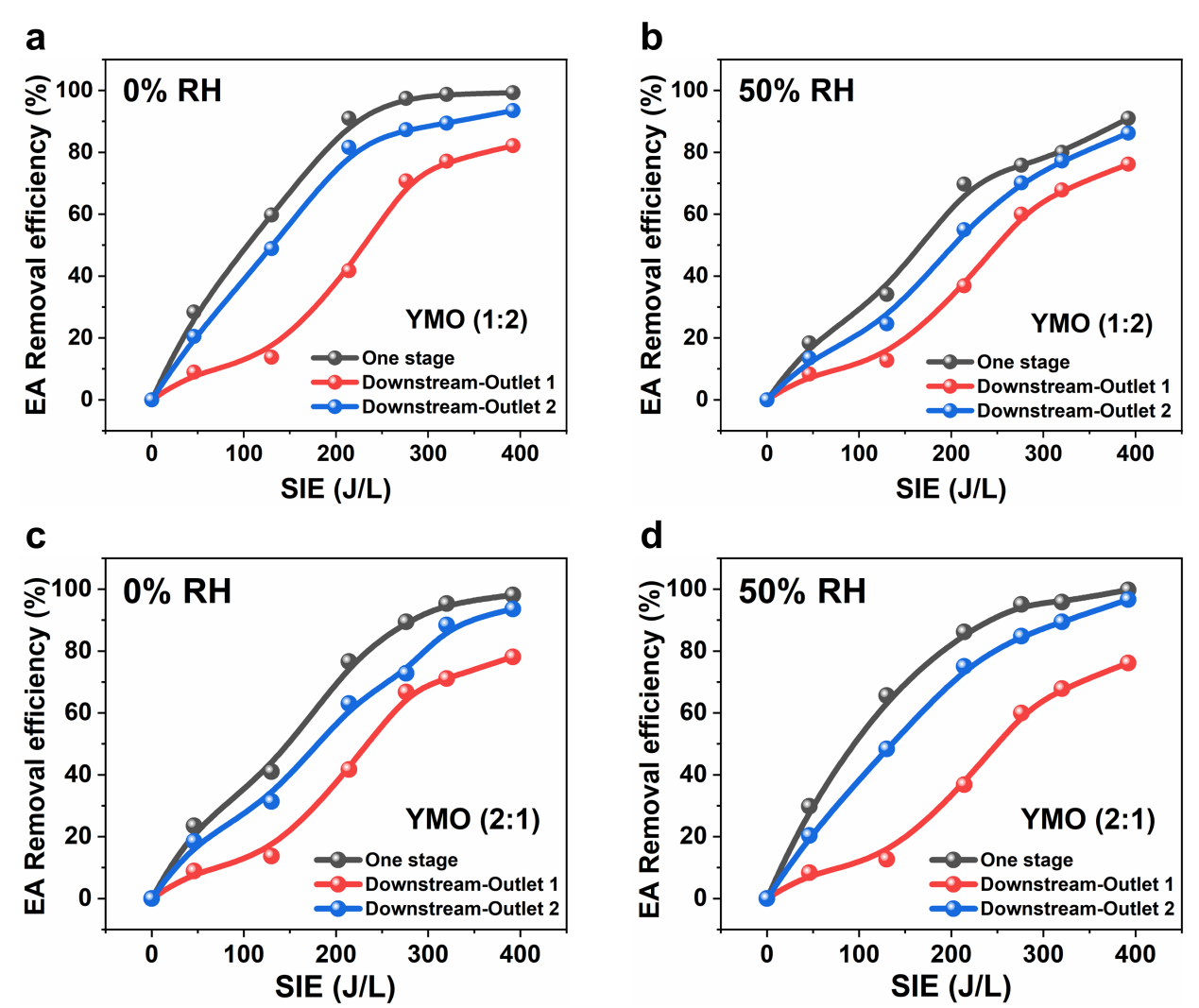


## **Figure S20.** Performance of (a-b) YMO (1:2) and (c-d) YMO (2:1) placed in plasma zone and downstream of the plasma under the relative humidity of 0% and 50%.

O_3_ is the only long-lived species in plasma with a half-life of 15~30 min^[7]^. It is observed that the outlet-2 exhibits much greater performance than outlet-1 for all samples in **Figure S20**. Besides, the performance of outlet is slightly better than that of outlet-2, demonstrating the synergy of plasma and catalysts. This suggests that the degradation and utilization of O_3_ make critical contributions to EA degradation.


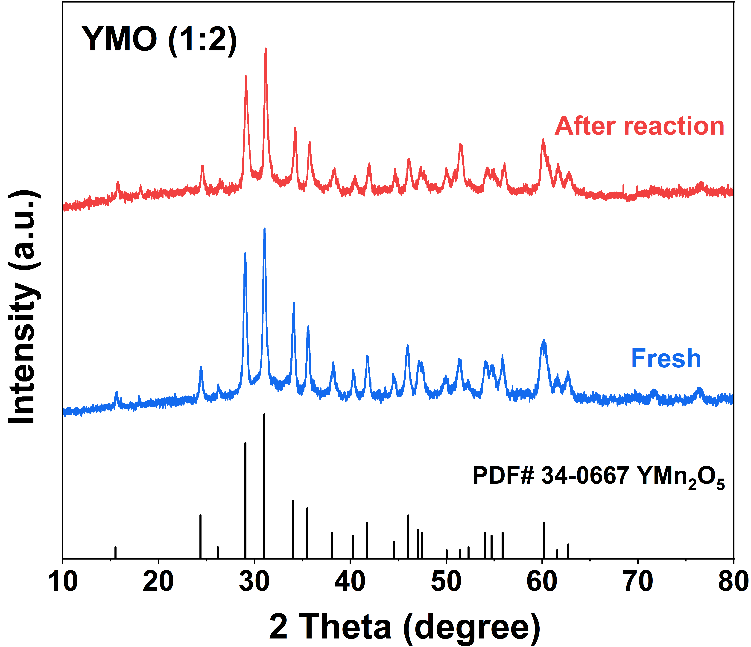


## **Figure S21.** XRD patterns for YMO (1:2) before and after long-time experiment.


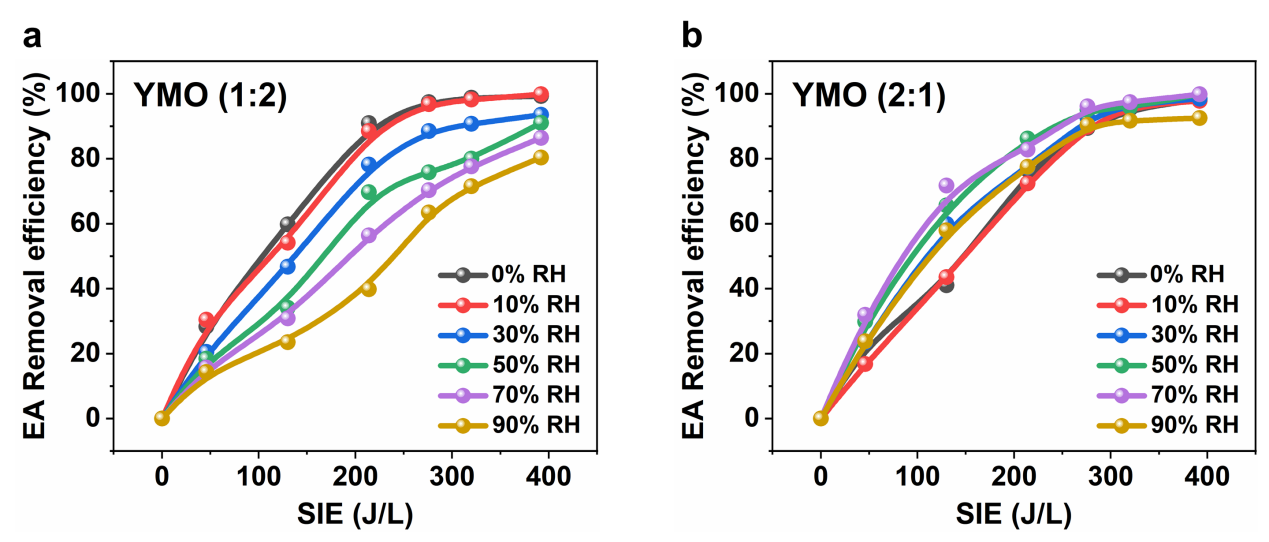


## **Figure S22**. EA removal efficiency with SIE of (a) YMO (1:2) and (b) YMO (2:1) sample under the relative humidity of 0~90%.


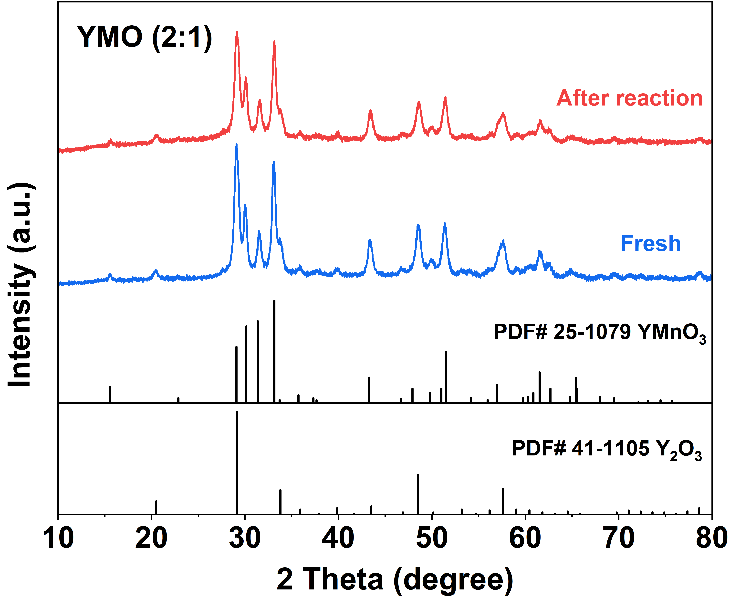


## **Figure S23.** XRD patterns for YMO (2:1) before and after long-time experiment.


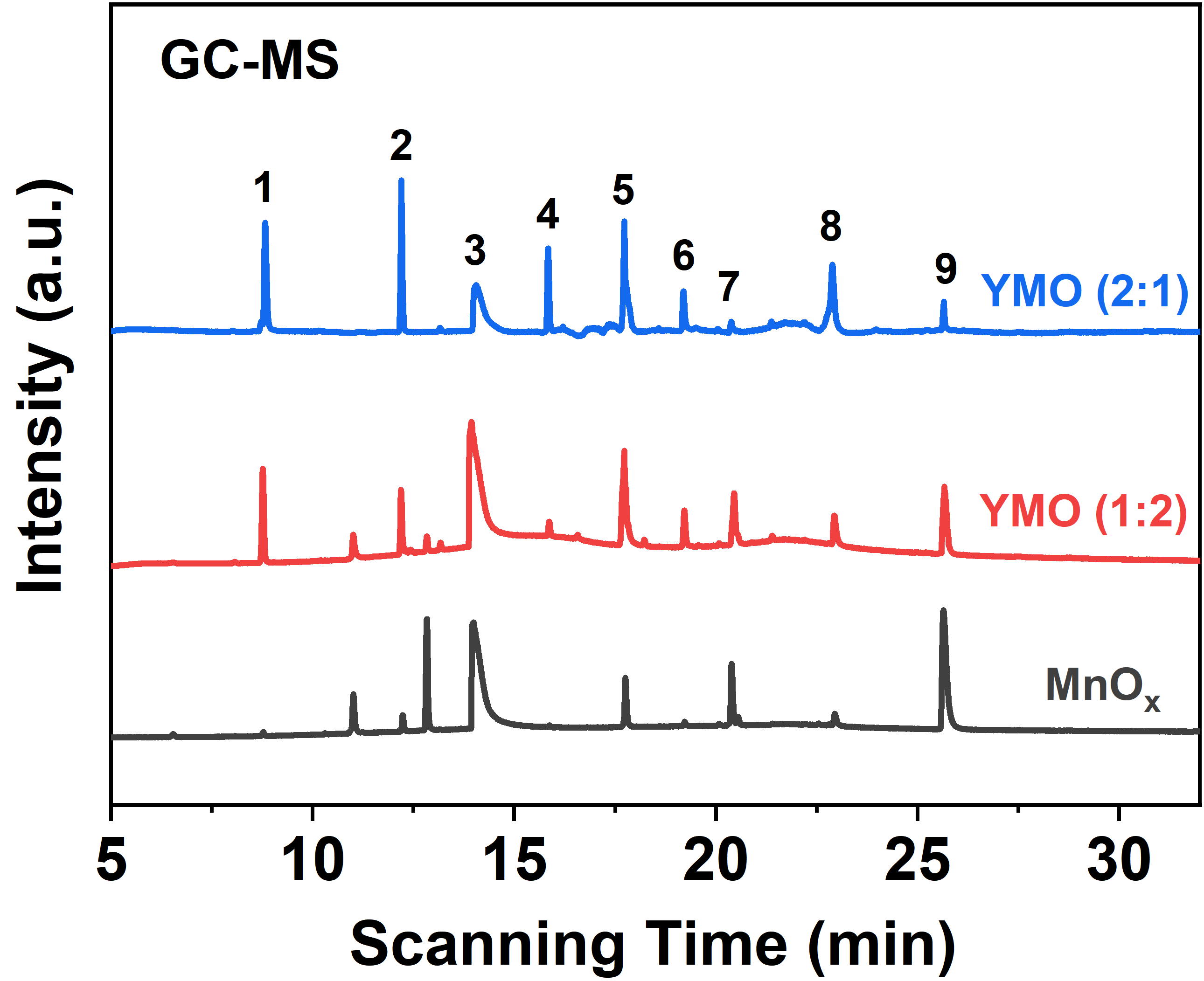


## **Figure S24.** The gas phase by-products detected by GC-MS.


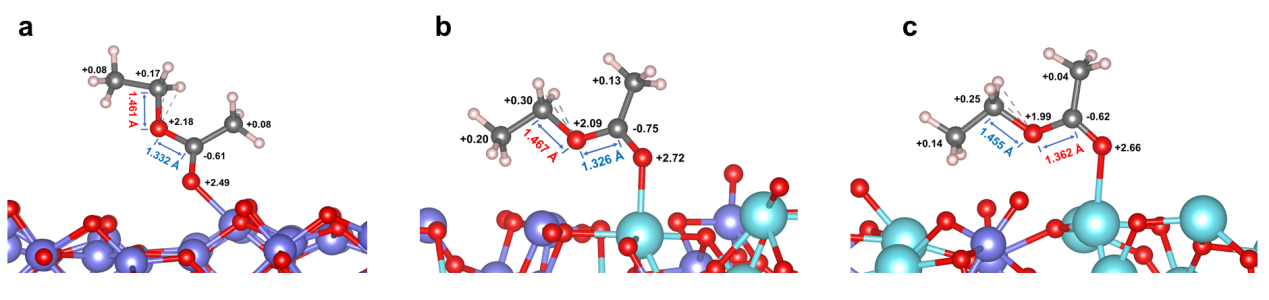


## **Figure S25.** The electron distribution and C-O bond length of the EA molecule adsorbed on (a) MnO_x_, (b) YMO (1:2) and (c) YMO (2:1).


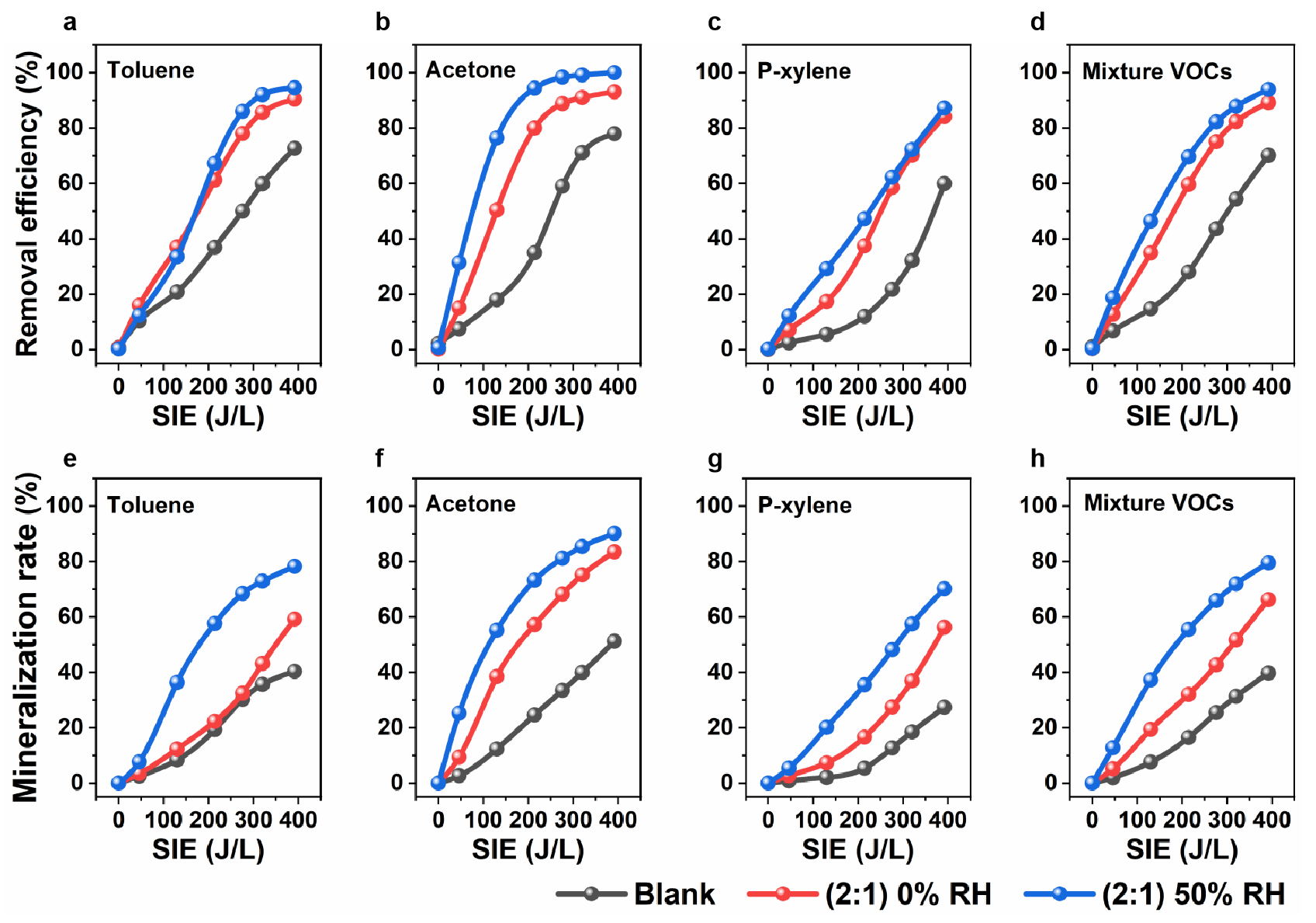


## **Figure S26**. The degradation of other typical VOCs: the removal efficiency and mineralization of (a, e) toluene (100 ppm), (b, f) acetone (100 ppm), (c, g) p-xylene (100 ppm) and (d, h) mixture VOCs (25 ppm ethyl acetate + 25 ppm toluene + 25 ppm acetone + 25 ppm p-xylene).

# Supplementary Tables

## **Table S1.** The results of XRD Rietveld refinements (performed with GSAS2 software)

| Sample | Phase composition (volume fraction) | Lattice parameters | | | |
| --- | --- | --- | --- | --- | --- |
|  |  | a (Å)  b (Å)  c (Å) | α(°)  β(°)  γ(°) | Volume(Å^3^) | Space group |
| MnO_x_ | Mn_3_O_4_ | 5.7566(17)  5.7566(17)  9.3997(31) | 90  90  90 | 311.49(27) | I41/amds |
| YMO (1:4) | YMn_2_O_5_ (62.68%) | 7.2665(12)  8.4641(13)  5.6692(8) | 90  90  90 | 348.68(12) | Pbam |
|  | Mn_3_O_4_ (37.32%) | 5.7628(10)  5.7628(10)  9.4694(18) | 90  90  90 | 314.48(14) | I41/amds |
| YMO (1:2) | YMn_2_O_5_ | 7.2740(16)  8.4602(16)  5.6669(10) | 90  90  90 | 348.74(17) | Pbam |
| YMO (1:1) | YMnO_3_ | 6.1545(6)  6.1545(6)  11.3313(11) | 90  90  120 | 371.71(7) | P63cm |
| YMO (2:1) | YMnO_3_ (65.70%) | 6.1603(87)  6.1603(87)  11.4052(155) | 90  90  120 | 383.97(95) | P63cm |
|  | Y_2_O_3_ (34.30%) | 10.6035(53)  10.6035(53)  10.6035(53) | 90  90  90 | 1192.19(1.83) | Ia-3 |
| YMO (4:1) | YMnO_3_ (42.48%) | 6.1690(12)  6.1690(12)  11.4000(25) | 90  90  120 | 375.729(11) | P63cm |
|  | Y_2_O_3_ (57.52%) | 10.5879(8)  10.5879(8)  10.5879(8) | 90  90  90 | 1186.94(28) | Ia-3 |

## **Table S2.** FTIR band assignments for Figure S8.

| Wavenumber (cm^-1^) | Assignment | References |
| --- | --- | --- |
| 603 | Mn-O | [8] |
| 486 | O-Mn-O | [8b] |
| 576 | Y-O | [8a] |
| 1084 | Y-OH | [9] |
| 1632 | chemisorbed water | [10] |
| 3376 | O-H stretching | [10] |

## **Table S3.** GC-MS results for the as-prepared samples.

| Label | Molecular formula | Name of compound |
| --- | --- | --- |
| 1 | C_4_H_8_ | 2,methyl Propylene |
| 2 | C_2_H_4_O | Acetaldehyde |
| 3 | C_4_H_4_O | Furan |
| 4 | C_3_H_6_O | Acetone |
| 5 | CH_2_O_2_ | Formic acid |
| 6 | CH_3_NO_2_ | Methane, nitro- |
| 7 | C_4_H_8_O_2_ | Ethyl acetate |
| 8 | CH_2_O_2_ | Formic acid |
| 9 | C_2_H_4_O_2_ | Acetic acid |

# References

1. K. Cao, G. Guo, D. Vanderbilt, L. He, *Phys. Rev. Lett.* **2009**, *103*(25), 257201.
2. G. Zhu, W. Zhu, Y. Lou, J. Ma, W. Yao, R. Zong, Y. Zhu, *Nat. Commun.* **2021**, *12*(1), 4152.
3. S. Diodati, A. Minelli, P. Dolcet, S. Gross, *Surf. Sci. Spectra* **2015**, *22*(1), 1-20.
4. J. He, Y. Wang, X. Li, J. Xiao, Y. Liu, H. Li, N. Li, Q. Xu, D. Chen, J. Lu, *Chem. Eng. J.* **2023**, *475*, 146205.
5. B. Li, Q. Yang, Y. Peng, J. Chen, L. Deng, D. Wang, X. Hong, J. Li, *Chem. Eng. J.* **2019**, *366*, 92-99.
6. a) X. Xu, *Thin Solid Films* **2001**, 390(1-2), 237-242; b) R. Li, Q. Tang, S. Yin, T. Sato, *Fuel Process. Technol.* **2006**, *87*(7), 617-622.
7. M. Wu, C. Liu, C. Chiang, Y. Lin, Y. Lin, Y. Chang, J. Wu, *IEEE T. Plasma Sci.* **2018**, *47*(2), 1100-1104.
8. a) J. Shukla, M. Varshney, A. Mishra, *Mater. Today: Proc.* **2021**, *47*, 652-655; b) Y. Wang, H. Tian, *Optik.* **2020**, *201*, 163524.
9. L. Giang, T. Anh, L. Marciniak, D. Hreniak, W. Strek, W. Lojkowski, *Physics Procedia.* **2015**, *76*, 73-79.
10. H. Devi, W. Singh, R. Loitongbam, *J. Fluoresc.* **2016**, *26*, 875-889.
